# Genome Mining for Radical SAM Protein Determinants Reveals Multiple Sactibiotic-Like Gene Clusters

**DOI:** 10.1371/journal.pone.0020852

**Published:** 2011-07-08

**Authors:** Kiera Murphy, Orla O'Sullivan, Mary C. Rea, Paul D. Cotter, R. Paul Ross, Colin Hill

**Affiliations:** 1 Microbiology Department, University College Cork, Cork, Ireland; 2 Teagasc Food Research Centre, Moorepark, Fermoy, County Cork, Ireland; 3 Alimentary Pharmabiotic Centre, Cork, Ireland; Emory University, United States of America

## Abstract

Thuricin CD is a two-component bacteriocin produced by *Bacillus thuringiensis* that kills a wide range of clinically significant *Clostridium difficile*. This bacteriocin has recently been characterized and consists of two distinct peptides, Trnβ and Trnα, which both possess 3 intrapeptide sulphur to α-carbon bridges and act synergistically. Indeed, thuricin CD and subtilosin A are the only antimicrobials known to possess these unusual structures and are known as the sactibiotics (**s**ulplur to **al**pha **c**arbon-containing an**tibiotics**). Analysis of the thuricin CD-associated gene cluster revealed the presence of genes encoding two highly unusual SAM proteins (TrnC and TrnD) which are proposed to be responsible for these unusual post-translational modifications. On the basis of the frequently high conservation among enzymes responsible for the post-translational modification of specific antimicrobials, we performed an *in silico* screen for novel thuricin CD–like gene clusters using the TrnC and TrnD radical SAM proteins as driver sequences to perform an initial homology search against the complete non-redundant database. Fifteen novel thuricin CD–like gene clusters were identified, based on the presence of TrnC and TrnD homologues in the context of neighbouring genes encoding potential bacteriocin structural peptides. Moreover, metagenomic analysis revealed that TrnC or TrnD homologs are present in a variety of metagenomic environments, suggesting a widespread distribution of thuricin-like operons in a variety of environments. *In-silico* analysis of radical SAM proteins is sufficient to identify novel putative sactibiotic clusters.

## Introduction

The ever-increasing issue of bacterial resistance to conventional antibiotics and consumer demands for safe minimally processed foods have stimulated research interest in natural antimicrobial agents such as bacteriocins [1], [2], [3]. A variety of approaches can be employed to identify novel bacteriocins with the most traditional being the isolation of strains and a culture-based assessment of their ability to produce novel antimicrobials which have a broad antimicrobial spectra. In contrast, the discovery of thuricin CD was the result of the specific mining of the gut microbiota for potentially therapeutic bacteriocins with a narrow spectrum of activity [4]. This alternative approach stems from the fact that in the clinical setting the use of broad spectrum antimicrobials has lead to the emergence of multi-drug resistant pathogens and the observation that the disruption of the colonic microbiota through administration of broad spectrum antibiotic treatment predisposes susceptible individuals to infection by the nosocomial pathogen *Clostridium difficile* [5], [6]. Thuricin CD, produced by the human faecal isolate *Bacillus thuringiensis* DPC 6431, is a two peptide antimicrobial, composed of Trnα and Trnβ. The two thuricin CD peptides act synergistically, exhibiting a narrow spectrum of activity targeting mainly the spore forming genera - *Bacillus* and *Clostridium*. Interestingly thuricin CD kills a wide range of clinical *C. difficile* ribotypes commonly associated with *Clostridium difficile* associated diarrhoea (CDAD) including the hypervirulent strain B1/NAP1/027 [7]. In a distal colon model thuricin CD, while killing *C. difficile,* had no significant impact on the composition of the microbiota unlike other broad spectrum antimicrobials [8]. However to be used as an oral therapeutic for CDAD thuricin CD, because it is sensitive to proteolytic enzymes, will require the use of encapsulation technologies to ensure the delivery of biologically active peptides to the colon.

Both Trnα and Trnβ are post-translationally modified peptides which contain unusual sulphur to α-carbon linkages, and as a result thuricin CD has been classified as a sactibiotic (**s**ulphur to **a**lpha **c**arbon an**tibiotic** [9]). The thuricin CD-associated gene cluster has been identified and contains genes, *trnC* and *trnD*, predicted to encode two radical S'-adenosylmethionine (SAM) proteins. Radical SAM proteins are characteristically associated with unusual posttranslational modifications and are likely to be involved in the formation of the aforementioned cysteine to α-carbon linkages [10]. Radical SAM-encoding genes are rare in bacteriocin-associated clusters, with subtilosin A and propionicin F being notable exceptions [11], [12]. Propionicin F is not a modified peptide and in this instance it is thought that the associated radical SAM enzyme contributes to N-terminal cleavage [12]. However, subtilosin A is a cyclic peptide which undergoes posttranslational modification involving a thiol linkage of sulphur to α-carbon and can therefore be classified as a sactibiotic. The mechanism associated with the formation of this unusual posttranslational modification has not been established but is thought to be due to the associated radical SAM enzyme, AlbA.

An alternative approach to the identification of novel post-translationally modified peptides has involved *in silico* screening to identify the most highly conserved component of the associated gene clusters i.e. the genes encoding the modification enzymes. This approach has been employed, for example, to identify novel type I and type II lantibiotics [13], [14], [15], [16] and thiazole/oxazole-modified microcin (TOMMS; [17], [18], [19], [20], [21]). Here this approach has been adapted to identify novel thuricin CD-like gene clusters. More specifically, an *in silico* screen of databases was undertaken, using TrnC and TrnD as driver sequences, to identify similar gene clusters. The search revealed 100 TrnC homologs and 53 TrnD homologs, which upon further investigation led to the identification of 15 novel putative thuricin CD-like clusters, i.e. clusters containing genes encoding TrnC and TrnD homologs as well as at least one structural peptide, across three phyla.

## Results

### *In silico* screen for TrnC and TrnD Protein

The sequences of TrnC and TrnD, radical SAM proteins encoded within the thuricin CD gene cluster [4], were used as driver sequences to perform a homology search against the complete non-redundant database (November 2010). The search revealed 100 TrnC homologs and 54 TrnD homologs (homology being defined as BLASTP E-value <10^-6^), in 112 unique genomes. Tables S1 and S2 provide a full list of TrnC and TrnD homologs, which were found in the genomes of sequenced microorganisms. In each case the corresponding bacterial genomes were viewed and the regions containing the genes of interest were located. None of the strains identified have previously been associated with a bacteriocin producing phenotype.

A more detailed examination of these gene clusters involved an analysis of the regions flanking the *trnC* and *trnD* determinants for other open reading frames (ORFs) potentially involved in the biosynthesis of, or immunity to, bacteriocins. The presence of a second nearby Radical SAM gene (including those encoding another TrnC or TrnD homolog) was screened for in each case. Putative Radical SAM proteins were examined for the presence of signature motifs. Enzymes of the Radical SAM superfamily generate radicals by combining a 4Fe-4S cluster and S-adenosylmethionine (SAM) in close proximity. The Radical SAM signature motif, C-X_3_-C-X_2_-C, which coordinates the iron-sulphur and SAM in close proximity was screened for, as were the putative SAM binding sequence motifs, GGEPLL and TNG.

Particular focus was placed on screening the aforementioned clusters for genes potentially encoding structural peptides which, given the variable nature of the structural peptides encoded within modified bacteriocin associated gene clusters, may or may not resemble the very similar Trnα and Trnβ peptides. One feature that was screened for was the presence of a conserved double-glycine (GG) motif, which is a common feature among bacteriocins that are synthesized as biologically inactive precursor peptides (prepeptides) and plays a key role in secretion and activation [22], [23]. Interestingly, in thuricin CD cleavage occurs between the GG residues rather than after which is the norm. The Trnα and Trnβ peptides consist of a cysteine-less leader region characterised by a high frequency of Glu and Asp residues [4]. Both genes are preceded by perfect ribosomal binding sequences (RBS) identical to that of the Shine-Dalgarno consensus that are embedded within identical stretches of nucleotides (AAAAATAAGGAGGAATTATC). Also screened for was the presence of conserved cysteine residues such as those that occur at positions +5, +9 and +13 in Trnα and Trnβ, forming a CX3CX3C formation. Cys +5 is flanked by small hydrophobic amino acids Ala and Val, and those at positions +9 and +13 are coupled with variant small hydrophobic residues. A conserved Ser residue is present at position +15. Additionally, Gly residues occur at positions +17 and +19, which are followed by variant hydrophobic and hydrophilic residues, respectively [4].

Sequencing had revealed that Trnα and Trnβ are post-translationally modified at their respective 21^st^, 25^th^ and 28^th^ residues [4]. It is believed that each peptide has three sulphur to α-carbon thioether linkages between the cysteine side chains and the α-carbons of the modified residues. These unusual sulphur to α-carbon linkages have also been reported in the antimicrobial subtilosin A [24]. Notably, a SAM-containing enzyme is thought to be responsible for forming the thioether linkages in subtilosin A [24]. This protein bears some resemblance to the SAM-containing enzymes encoded within the thuricin CD operon [4]. Other features that were specifically screened for were the presence of other genes predicted to encode bacteriocin associated proteins, including those involved in transport and processing.

**Figure 1:**
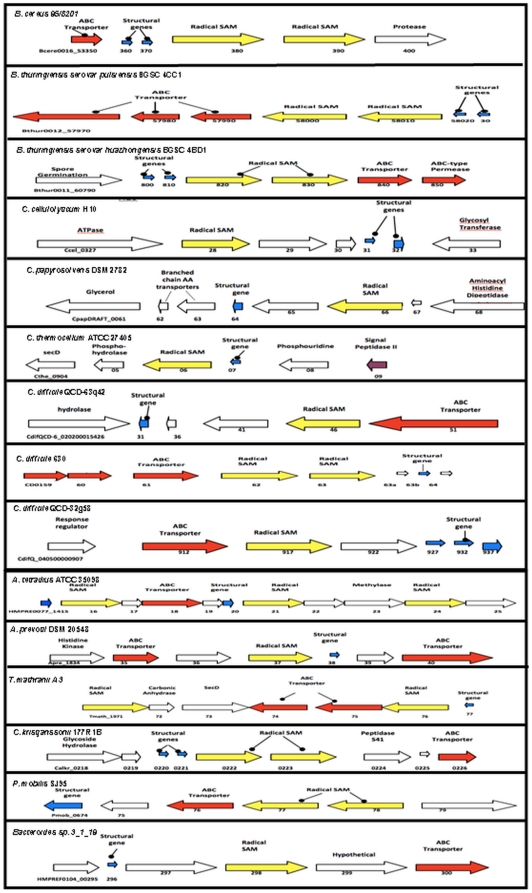
Diagrammatic representation of the thuricin CD-like gene clusters found in the TrnC and TrnD screens. Genes involved in transport are coloured red, structural genes in blue, radical SAM encoding genes in yellow and genes involved in spore germination in green.

Following this screen, 15 gene clusters from 15 different bacterial strains were deemed worthy of closer analysis. Of these, 10 were gene clusters that contained both TrnC and TrnD encoding genes with putative prepeptide-encoding genes. The remaining 5 contained either a TrnC or a TrnD encoding gene but were deemed noteworthy as the majority of the other thuricin CD operon-associated features were present. Thirteen of these 15 total gene clusters were in the genomes of strains within the phylum Firmicutes. The other 2 strains were *Bacteroides* sp. 3_1_19 of the phylum Bacteroidetes and *Petrotoga mobilis* SJ95 of the phylum Thermotogae. These 15 clusters are described below and are grouped according to the phylum and genus of the associated strain. Arrow diagrams representing each of these clusters are depicted in Figure 1.

### Gene clusters in Firmicutes

#### Identification of novel Bacillus-associated bacteriocin gene clusters

*Bacillus* is a large and diverse genus which contains both free living and pathogenic species. Members are Gram-positive, rod-shaped, sporulating and are known to produce a wide range of antimicrobial substances including bacteriocins. Many of the *Bacillus* bacteriocins are lantibiotics, a category of post-translationally modified peptides which are grouped as a consequence of the presence of (β-methyl) lanthionine structures. Examples include subtilin [25], ericin S and ericin A [26], haloduracin [14], [16] and lichenicidin [13]. Our *in silico* screen highlighted the presence of thuricin CD-like gene clusters in the genomes of three *Bacillus* strains, i.e. *B. cereus* 95/8201, *B. thuringiensis serovar pulsiensis* BGSC 4CC1 and *B. thuringiensis serovar huazhongensis* BGSC 4BD1.

#### *B. cereus* 95/8201

Strains of *B. cereus* are frequently isolated from soil but also found in foods of plant and animal origin. *B. cereus* is an opportunistic pathogen and has frequently been associated with human food poisoning [27].

*B. cereus* strain 95/8201 (GenBank accession ACMF00000000) was isolated from a human patient with endocarditis (*Bacillus cereus* group Tourasse-Helgason MLST Database) and sequenced by the Naval Medical Research Centre in 2009. A gene cluster was identified in this strain which is very similar to the thuricin CD gene cluster in *B. thuringiensis* DPC 6431. Bcere0016_53380 (499 residues) resembles TrnC (506 amino acids). The C-X_3_-C- X_2_-C motif occurs as CNLRCEYC at Cys107 in Bcere0016_53380 and is identical to the corresponding region in TrnC. Putative SAM binding sequence motifs, GGEPLL (identified at Gly165) and TNG (identified at Thr195) are also conserved between Bcere0016_53380 and TrnC. Bcere0016_53390 (408 amino acids) resembles TrnD (414 residues). The signature motif occurs as CMNMCKHC (at Cys103 in Bcere0016_53390) in both. The GGEPFL (Gly158) and TNG (T187) motifs in Bcere0016_53390 are identical to those in TrnD. Bcere0016_53360 and Bcere0016_53370 are the predicted structural genes and are 100% identical to *B. thuringiensis* DPC 6431 structural genes, trnβ and trnα respectively. Other genes of note in this cluster are those encoding a putative ABC transporter (Bcere0016_53350) and a C-terminal protease (Bcere0016_53400). The ABC transporter may be responsible for the export of the bacteriocin from the cytoplasm and the peptidase may have a role in immunity or leader cleavage.

#### *B. thuringiensis serovar pulsiensis* BGSC 4CC1

*B. thuringiensis* are noted for their ability to produce a parasporal crystal upon sporulation. Due to its insecticidal properties it has been used commercially as a biological insecticide [28]. *B. thuringiensis serovar pulsiensis* BGSC 4CC1 (Accession NZ_ACNJ00000000) was sequenced by the Naval Medical Research Centre in 2009. Within this genome, putative TrnC (Bthur0012_58010) and TrnD (Bthur0012_58000) encoding genes were identified which are surrounded by other genes associated with bacteriocin synthesis. The TrnC homolog (e-value 5e-30) encoded by Bthur0012_58010 contains the GGEPLL motif at Gly35 while the TrnD homolog (e-value 8e-23) encoded by Bthur0012_58000 has a GGDPLL at Gly167 and a C-X_3_-C- X_2_-C motif (CNKNCIHC) at Cys116. Bthur0012_58020 and Bthur0012_58030 appear to be two identical structural genes consisting of 47 amino acids. However, whilst they have a C-X_3_-C- X_3_-C motif (CALKCAGPC) at Cys12 the GG motif is at Gly44, making the resulting peptide only 3 peptides long. This suggests that there may be alternative structural peptides in this operon we have failed to recognise or alternatively cleavage in this peptide may occur at a different site. Surrounding genes of note encode three ABC transporters (Bthur0012_57970, Bthur0012_57980 and Bthur0012_57990).

#### *B. thuringiensis serovar huazhongensis* BGSC 4BD1

*B. thuringiensis serovar huazhongensis* BGSC 4BD1 (Accession NZ_ACNI00000000) was also sequenced by the Naval Medical Research Centre in 2009. The thuricin CD-like gene cluster identified here is very similar to those found in the other *Bacillus* species listed above. The BGSC 4BD1-associated cluster contains two putative Radical SAM encoding genes, Bthur0011_60820 and Bthur0011_60830. Bthur0011_60820 encodes a TrnC homolog (e-value 6e-30), contains 359 amino acids and a GGEPLL motif at Gly35. The TrnD homolog (e-value 2e-23), encoded by Bthur0011_60830, is 409 amino acids, has a GGDPLL motif at Gy167 and a TNG motif at Thr198. Interestingly, the two predicted structural genes, Bthur0011_60810 and Bthur0011_60800 are identical to those found in *B. thuringiensis serovar pulsiensis* BGSC 4CC1 described above. Adjacent genes potentially of relevance are predicted to encode an ABC-type transporter (Bthur0011_60840) and a permease (Bthur0011_60850).

### Identification of novel *Clostridium*- and *Clostridia*-associated bacteriocin gene clusters

*Clostridium* (family *Clostridiaceae*) is a genus of gram positive, anaerobic, endospore-forming, Firmicutes, of which there are approximately 100 species. These include important pathogens such as *C. difficile, C. perfringens* and *C. tetani.* Several *Clostridium*-associated bacteriocins have been characterised from this genus, including boticin B [29] and bacteriocin 28 [30]. Bioinformatic analysis revealed the presence of thuricin CD-like clusters in genomes of the genus *Clostridium*, specifically within the genomes of, *C. cellulolyticum* H10, *C. papyrosolvens* DSM 2782, *C. thermocellum* ATCC 27405 *C. difficile* QCD-63q42, *C. difficile* 630 and *C. difficile* QCD-32g58. Four further clusters were identified within the broader class *Clostridia*. They included two clusters identified in the genus *Anaerococcus* (family *Curculionoidea*); *Anaerococcus tetradius* ATCC 35098 and *Anaerococcus prevotti* DSM 20548-plasmid pAPRE01. Thuricin CD like clusters were also identified in *Thermoanaerobacter mathranii A3* (family *Thermoanaerobacteraceae*) and *Caldicellulosiruptor kristjanssonii* 177R1B (family *Thermoanaerobacterales*).

#### *C. cellulocyticum* H10

*C. cellulocyticum* H10 (Accession YP_002504697) is a mesophilic cellulolytic bacterium that was originally isolated from decayed grass compost [31]. The ability of this species to degrade cellulose is an active area of interest as the products of this degradation, ethanol and hydrogen, may be used an alternative form of energy [32]. The *C. cellulocyticum* H10 sequence was published in 2009 by US DOE Joint Genome Institute. Within the thuricin CD-like cluster of this strain, Ccel_0328 encodes an apparent TrnC homolog (e-value 7e-30). This 486 amino acid protein contains a GGEPLL motif at Gly145, a TNG motif at Thr177 and a C-X_3_-C-X_2_-C motif, CNLRCNYC, at Cys94. Ccel_0328 was also predicted to encode a TrnD homolog (e-value 2e-6). An adjacent gene also expressed low level homology with TrnD (e-value 0.81). Two possible structural genes, Ccel_0331 and Ccel_0332, were identified. These are predicted to encode peptides 32 amino acids and 63 amino acids in length, respectively. Both contain GG motifs and cysteine residues, albeit not in the C-X_3_-C-X_3_-C formation. Other adjacent genes are predicted to encode a glycosyl transferase, Ccel_0333 and an AAA-type ATPase (ATPases associated with diverse cellular activities), Ccel_0327.

#### *C. papyrosolvens* DSM 2782

*C. papyrosolvens* DSM 2782 (Accession NZ_ACXX00000000) was isolated from intertidal mud from the Don River in Scotland [33] and sequenced in 2009 by the US DOE Joint Genome Institute. The gene cluster of note examined here contains a putative TrnC (482 amino acid) encoding gene, CpapDRAFT_0066 (e-value 4e-28). CpapDRAFT_0066 has a GGEPLL at Gly154 and a C-X_3_-C-X_2_-C (CNLRCKYC) motif at Cys103. The protein (375 amino acids) encoded by the adjacent gene bears some slight resemblance to TrnD (e-value 2.8). CpapDRAFT_0064 may encode a structural peptide with a GG motif at Gly78. Other bacteriocin-associated genes include those encoding a dipeptidase (CpapDRAFT_0068) and two branched chain amino acid transporters (CpapDRAFT_0062 and CpapDRAFT_0063).

#### *C. thermocellum* ATCC 27405

*C. thermocellum* is a thermophilic, cellulolytic bacterium capable of directly converting cellulosic substrate into ethanol. The ATCC 27405 strain (Accession NC_009012) was sequenced in 2007 by the US DOE Joint Genome Institute. While this particular gene cluster only contains one Radical SAM protein and one putative structural peptide encoding gene, the high similarity of their sequences to those found in the other clusters is noteworthy. As a consequence the cluster was the focus of closer inspection. The Radical SAM-encoding gene identified, Cthe_0906, resembles both TrnC (e-value 3e-16) and TrnD (e-value 3e-5). This protein contains 450 amino acids, a GGEPMM motif at Gly153 and a TNG motif at Thr87. A C-X_3_-C-X_2_-C motif, namely CNLRCKYC, is identified at Cys104. Interestingly, the CNLRCKYC sequence was also observed in Calkr_0222, a TrnC homolog in *C. kristjanssonii* 177R1B. Cthe_0907 is believed to encode a structural gene. It is 46 amino acids long, contains a GG motif at Gly19 and a C-X_3_-C-X_3_-C motif (CQTSCQSAC) at Cys24. Within this cluster other genes potentially of relevance include *secD*, a gene involved in protein export (Cthe_0904) and also a signal peptidase-encoding gene (Cthe_0909).

#### *C. difficile* QCD-63q42

*C. difficile* is the leading cause of nosocomial infections associated with antibiotic use and is responsible for substantial morbidity and mortality worldwide [34]. *C. difficile* QCD-63q42 is one of three *C. difficile* strains in which thuricin CD-like gene clusters were identified in this study. *C. difficile* QCD-63q42 (Accession NZ_ABHD00000000) was isolated from a 67 year old male patient with severe *C. difficile* Associated Disease (CDAD) and was sequenced in 2007 by McGill University. Like the aforementioned *C. thermocellum* ATCC 27405 gene cluster, the QCD-63q42 gene cluster is lacking a TrnD homolog and only has one structural gene but was included for the same reasons. The TrnC homolog encoded by CdifQCD-6_020200015426 (e-value 4e-21) contains 475 amino acids, a GGEPLL motif at Gly148 and the C-X_3_-C-X_2_-C motif (CNLHCDYC) at Cys97. The proposed structural peptide, encoded by CdifQCD-6_020200015431, is larger than those previously observed (79 amino acids) but does contain two GG motifs, at Gly 21 and Gly 39, and also a C-X_3_-C-X_3_-C motif, CGALCANLC, at Cys25. Nearby genes include those encoding a hydrolase (CdifQCD-6_020200015426) and an ABC transporter (CdifQCD-6_020200015451).

#### *C. difficile* 630

The complete genome sequence of C. difficile strain 630 (Accession NC_009089) was sequenced in 2006 by the Sanger Institiute [34]. The strain was isolated from a patient with severe pseudomembranous colitis in Switzerland and is a virulent, highly transmissible, drug resistant strain. CD0162 is a homolog of both TrnC and TrnD (e-values of 5e-30 and 2e-8, respectively). While the adjacent CD0163 was annotated as hypothetical, alignment of its sequence with TrnD confirmed it is a TrnD homolog (e-value 1e-12). Both CD0162 (473 amino acids) and CD0163 (356 amino acids) lack the signature C-X_3_-C- X_2_-C motif but both possess a GG motif.

CD0163B is thought to encode a potential structural gene of 67 amino acids containing a GG motif at Gly47 and a C-X_3_-C-X_3_-C motif (CTIMCPYTC) at Cys20. This gene cluster also contains genes that code for an ABC transporter (CD0161) and a two-component response regulator (CD0159 and CD0160).

#### *C. difficile* QCD-32g58

*C. difficile* QCD-32g58 (Accession NZ_AAML00000000) was sequenced in 2007 by Washington University. This hyper-virulent strain was responsible for a multi-institutional outbreak of CDAD in Quebec, Canada [35]. Analysis of this genome revealed the presence of a TrnC homolog-encoding gene, CdifQ_040500000917 (e-value 6e-30). The corresponding protein contains a GGEPLL motif at Gly153 and a C-X_3_-C-X_2_-C motif, CNLRCDYC. As is the case with some of the other *Clostridia*-associated clusters strains examined, an adjacent gene, CdifQ_040500000922, was found to encode a protein (356 amino acids) that does not significantly resemble TrnD (e-value 4.5). A screen for potential structural genes revealed the presence of three adjacent genes of relevant size. Upon further analysis, the 67 amino acid CdifQ_040500000932 was determined to be most likely to be the structural peptide-encoding gene as its product is predicted to contain a GG motif at Gly47 and a C-X_3_-C-X_3_-C motif, CTIMCPYTC at Cys20. Surrounding genes of note include those encoding an ABC transporter (CdifQ_040500000912) and a response regulator (CdifQ_040500000907).

#### *Anaerococcus tetradius* ATCC 35098

*A. tetradius* is a strictly anaerobic bacterium, usually found in fresh water and hot springs. Lantibiotics have previously been associated with related microbes, such as ruminococcin A produced by *Ruminococcus gnavus* [36]. *A. tetradius* ATCC 35098 (Accession ACGC00000000) was sequenced in 2009 by Baylor College of Medicine. The gene cluster of interest within this genome may contain as many as 11 genes, including a number of apparently bacteriocin-associated genes. BLAST searches revealed that the proteins encoded by HMPREF0077_1416 (e value 3e-17) and HMPREF0077_1424 (e value 3e-23) are TrnC homologs. HMPREF0077_1416 contains 728 amino acids, a GGEPLL motif at Gly79, a TNG at Thr117 and a C-X_3_-C- X_2_-C motif (CNLRCSYC). HMPREF0077_1424 also contains a GGEPLL motif at Gly151, a TNG at Thr181 and CNLKCKYC at Cys104 and has 457 amino acids. Another protein annotated as a Radical SAM, HMPREF0077_1421, and containing 323 amino acids was examined. It contained a C-X_3_-C- X_2_-C motif, CNFSCLHC, at Cys18 but lacked GGEPLL and TNG motifs. A sequence alignment with TrnD showed a good agreement and gave an e value of 4e-4. While this is outside the threshold set at e-value <10^−6^, its presence remains noteworthy. In addition to the predicted radical SAM proteins, a hypothetical protein associated with this cluster shares homology with a methylase (HMPREF0077_1423). Methylases contribute to the production of a number of non-ribosomal antibiotics [37]. HMPRE0077_1413 and HMPRE0077_1414 encode homologues of the SalRK two component signal transduction system associated with the production of the lantibiotic salivaricin A2, produced by *Streptococcus salivarius* K12 [38]. In close proximity are genes encoding a response regulator (HMPREF0077_1414) and two ABC transporters (HMPREF0077_1418 and HMPREF0077_1426).

#### *A. prevotti* DSM 20548

*A. prevotii* is an anaerobic, mesophilic, non-motile, non-sporulating bacterium. It is an opportunistic pathogen found in the microflora of the mouth, skin or vagina of the host [39]. Strain DSM 20548 (Accession CP001709) was isolated from a human plasma sample and was sequenced in 2009 by the US DOE Joint Genome Institute. It contains a thuricin CD-like gene-cluster of interest on its plasmid pAPRE01. A putative TrnC (e-value 8e-37)-encoding gene, Apre_1837, was identified in this cluster. The corresponding 518 amino acid protein is predicted to contain a GGEPLI motif at Gly184 and a C-X_3_-C-X_2_-C motif, CNLRCKYC at Cys132. A CNLRCKYC motif was also observed in TrnC homologs encoded by gene clusters in *C. kristjanssonii* 177R1B, *C. papyrosolvens* DSM 2782 and *C. thermocellum* ATCC 27405. A hypothetical gene, Apre_1836, adjacent to the putative TrnC, of 426 amino acids was aligned with TrnD but no significant similarity was revealed. There are two potential structural genes, Apre_1838 and Apre_1839. Apre_1838 encodes a 65 AA protein, with a GA site at Gly14 and several cysteine residues. The product of Apre_1839 has a GG motif at Gly182 and C-terminal cysteines but is much larger than typical bacteriocin pre-peptides, consisting of 409 amino acids. The flanking region also contains genes encoding a histidine kinase (Apre_1834) and two ABC transporters (Apre_1835 and Apre_1840).

#### *Thermoanaerobacter mathranii* A3

*T. mathranii* A3 (Accession NC_014209) is a rod-shaped, motile, ethanol-producing, extremely thermophilic, anaerobic bacterium isolated from a hot spring in Iceland [40]. This strain was recently sequenced in 2010 by the US DOE Joint Genome Institute and contains a putative bacteriocin gene cluster. The *T. mathranii* A3 genome contains two radical SAM-encoding genes, the TrnC homolog-encoding Tmath_1971 (e value 3e-18) and the TrnD homolog-encoding Tmath_1976 (e value 3e-6). Tmath_1971 contains 461 residues, a GGEPLL at Gly153 and interestingly a C-X_3_-C- X_2_-C motif, namely CNLRCKYC, at Cys104, which has been observed in other TrnC homologs mentioned previously. Tmath_1976 is 453 amino acids long and contains a GGEPLL motif at Gly151, a TNG at Thr185 and a C-X_3_-C- X_2_-C motif, CNLKCEYC, at Cys101. A putative structural gene, Tmath_1977, was also identified. The corresponding peptide is predicted to contain 32 residues, a GG motif at Gly14 and two C-terminal cysteine residues. Other surrounding genes of note include those encoding two ABC transporters (Tmath_1974 and Tmath_1975) and *secD*, a gene involved in protein export (Tmath_1973).

#### *Caldicellulosiruptor kristjanssonii* 177R1B

*Caldicellulosiruptor* is a genus of anaerobic, extreme thermophilic bacteria [41]. *C. kristjanssonii* 177R1B (Accession NC_014721) was isolated from a hot spring biomat in Iceland and sequenced in 2010 by the US DOE Joint Genome Institute. The 177R1B thuricin CD-like gene cluster contains a number of thuricin CD-associated features. Calkr_0222 encodes a 492 amino acid TrnC homolog (e-value of 5e-77) that contains a GGEPLL motif at Gly172, a TNG motif at Thr193 and a C-X_3_-C- X_2_-C motif (CNLRCKYC) at Cys114. The TrnD homolog, encoded by Calkr_0223, contains 421 amino acids (e-value of 7e-61). A GGEPFI motif at Gly164, a TNG motif at Thr193 and a C-X_3_-C- X_2_-C motif (CNFDCKFC) at Cys110 were identified. Two possible structural peptides, encoded by Calkr_0221 and Calkr_0220, contain features associated with Trnβ and Trnα. Calkr_0221 contains 41 residues, a GG motif at Gly14 and a C-X_3_-C- X_3_-C motif (CWIGCGSFC) at Cys19. Calkr_0220 is 45 amino acids long and contains two possible GG cleavage sites at Gly14 and Gly25 and also a C-X_3_-C- X_2_-C motif (CLIACVGGC) at Cys19. There are also adjacent peptidase- (Calkr_0224) and ABC transporter- (Calkr_0226) encoding genes.

### Gene clusters in non-Firmicutes

The final two thuricin CD-like gene clusters of note were identified in strains that were non Firmicutes, i.e. *Petrotoga mobilis* SJ95 of the phylum Thermotogae phylum and *Bacteroides* sp. 3_1_19 of the phylum Bacteroidetes. These are described below.

#### *Petrotoga mobilis* SJ95

*P. mobilis* SJ95 (Accession NC_010003) is a rod-shaped, gram-negative, thermophilic bacterium from the order Thermotogales. It is able to reduce elemental sulphur to hydrogen sulfide and was isolated from the hot oilfield water of a North Sea oil reservoir [42]. There have been no reports to date of bacteriocin production by this species. Strain SJ95 was sequenced in 2007 by the US DOE Joint Genome Institute. A gene cluster of note was identified in this strain, which contains a number of potential bacteriocin-associated genes in close proximity to putative *trnC* and *trnD* determinants, Pmob_0678 (e-value 3e-36) and Pmob_0677 (e-value 1e-18) respectively. Pmob_0678 is a 464 amino acid protein with a GGEPLL motif at Gly136, a TNG motif at Thr168 and a C-X_3_-C-X_2_-C motif, CNLQCRYC at Cys78. Pmob_0677 contains 430 residues and the only observable motifs were a GG at Gly 173 and Gly332. A putative ABC transporter, Pmob_0676, was also observed nearby. The probable structural peptide is the 197 aa long hypothetical protein Pmob_0674, with GG motifs at Gly92 and Gly111, however it lacks any cysteine residues.

#### *Bacteroides* sp. 3_1_19

*Bacteriodes* are a genus of gram-negative, anaerobic, rod-shaped bacteria that are normal inhabitants of the oral, respiratory, intestinal, and urogenital cavities of humans and animals. Some species can cause potentially fatal abscesses and bacteremias [43]. *Bacteriodes* sp. 3_1_19 (Accession NZ_ADCJ00000000) was isolated from the gastrointestinal tract of a healthy 59-year-old female and was sequenced in 2010 by the The Broad Institute Genome Sequencing Platform. The genome of *Bacteriodes* sp. 3_1_19 contains a thuricin CD-like gene cluster. A TrnC homolog, encoded by HMPREF0104_00298, (e-value 2e-17) was identified. This 471 amino acid protein contains a GGEPLL motif at Gly143 and a C-X_3_-C- X_2_-C motif, CNLQCTYC, at Cys85. A potential TrnD-like protein, HMPREF0104_00299, which had been annotated as a hypothetical protein was examined further. Its size is 415 amino acids, has a GG motif at Gly183 and also has a C-X_3_-C- X_2_-C motif, CTFDCQHC, at Cys136. An alignment of this sequence to that of TrnD agreed well, with an e-value of 6e-5, and is likely to be a TrnD homolog. There are also genes encoding a predicted structural peptide (HMPREF0104_00296) with 54 residues, a GG motif at Gly27 and cysteine residues and an ABC transporter (HMPREF0104_00300).

### Metagenomic analysis

A search for putative TrnD and TrnC-encoding genes was also performed against metagenomic DNA. The search revealed 365 TrnC and 151 TrnD homologs in metagenomic databases (homology being defined as BLASTP e-value <10^6^) (Tables S3 and S4). This analysis revealed the presence of potential TrnC and TrnD-encoding genes in such diverse environments as the Indian Ocean [44], hypersaline lagoons from the Galapagos Islands, coastal sea water from the Gulf of Mexico [45], [46], farm soil from Waseca County, USA [47] and a coral reef from French Polynesia [44]. This search provides further insight into the diverse distribution of microorganisms potentially capable of producing thuricin CD-like products.

### Alignment of TrnC and TrnD- like Radical SAM proteins

**Figure 2:**
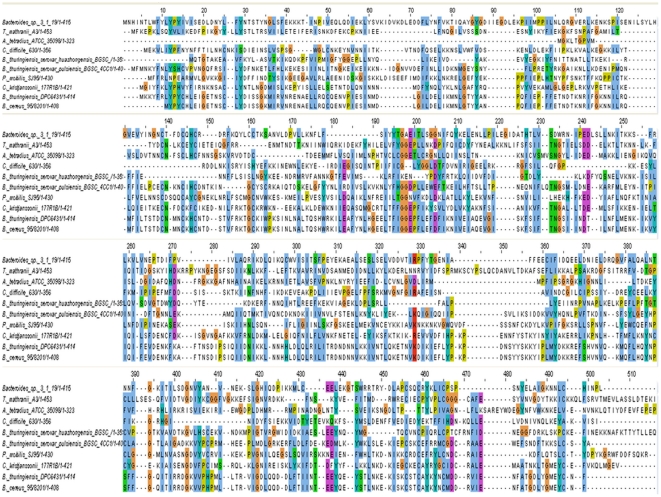
Protein sequence alignment of TrnD and all the TrnD homologs identified in this study.

**Figure 3:**
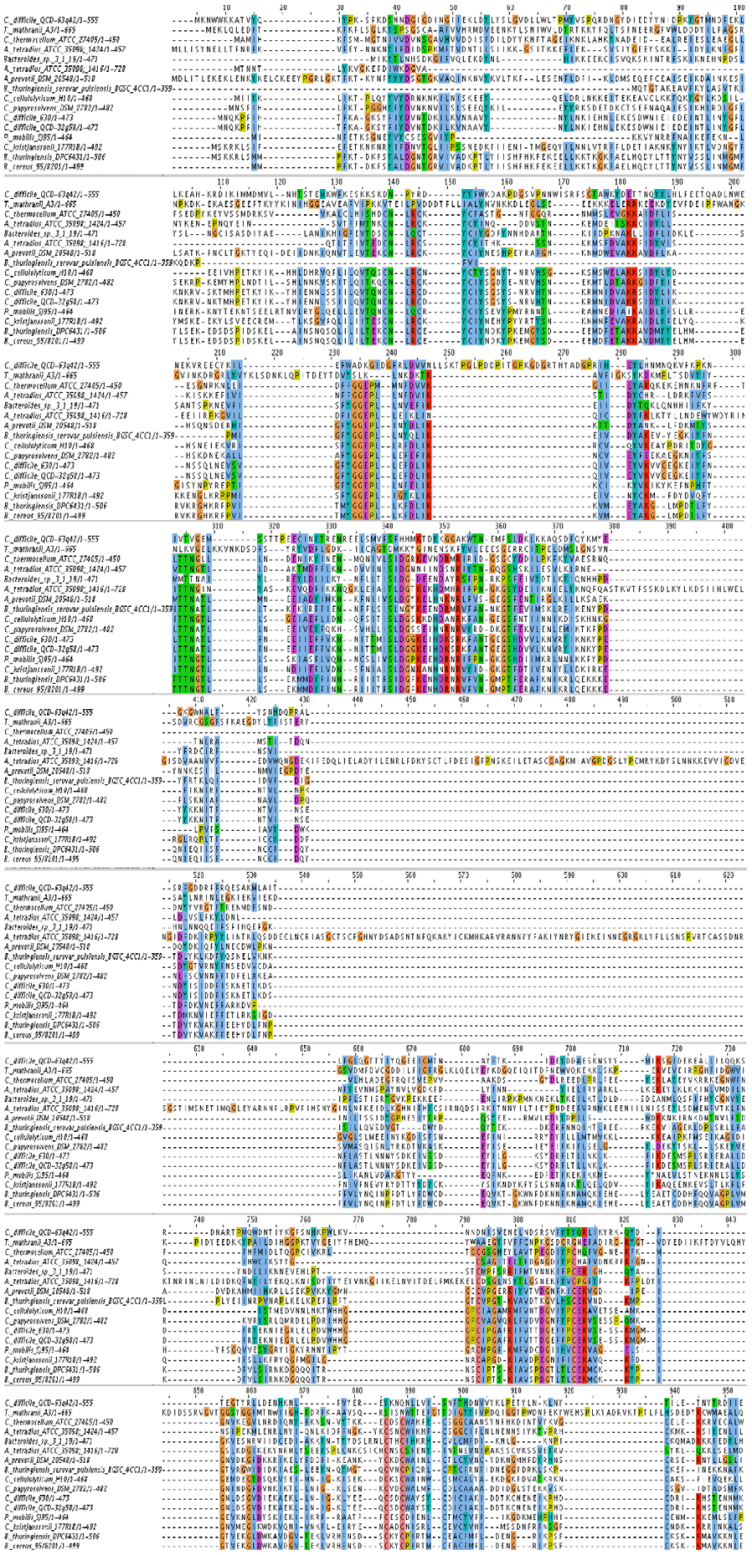
Protein sequence alignment of TrnC and all the TrnC homologs identified in this study.

The availability of a significant number of TrnC- and TrnD-like radical SAM protein sequences enabled further *in silico* analysis to identify conserved motifs and residues. Alignment of TrnD and 9 homologs (Fig. 2) reveals a number of completely conserved motifs and residues, which are summarized in Table 1. A radical SAM superfamily signature motif CX_3_-CX_2_-C was identified in 8 sequences. *B. thuringiensis serovar huazhongensis* BGSC 4BD1 Bthur0011_60830 and *C. difficile* 630 CD0163 did not contain this motif. Radical SAM enzymes generate catalytic radicals by combining a 4Fe-4S cluster and S-adenosylmethionine (SAM) in close proximity. The reaction requires the input of one electron, which is supplied by the 4Fe-4S cluster that is bound to the protein via cysteine ligands that reside in the CX_3_-CX_2_-C motif [48]. Kaminska *et al* [10] demonstrated that mutation of this motif abolishes activity of Cfr, a member of radical SAM superfamily which encodes a methyltransferase. Conserved cysteine residues are also located in a CX2-CX5-C conformation starting at alignment position 456. Another feature common to all radical SAM proteins is a glycine-rich sequence motif proposed to be the SAM-binding site. Both GGE/DP and TNG/A motifs were identified in 60% and 50% of radical SAM proteins respectively. These putative SAM binding sequence motifs have also been observed in AtsB, a Fe-S oxidoreductase arylsulfatase regulatory protein [49]. In addition to these, other motifs of note included a YD/N motif present in 60% of the aligned proteins, a CN motif also found in 60% of the alignments and a PC/S motif present in 50%. A number of conserved single residues were identified including a number of aromatic residues. Alignment of the TrnC homologs (Fig. 3) also revealed several conserved regions summarized in Table 2. As with the TrnD homologs, the heterogeneity of TrnC homologs is most pronounced within the C-terminal regions which are believed to be responsible for binding of substrates and auxiliary cofactors, whereas the sequences of the N-termini are well conserved. The characteristic CX_3_-CX_2_-C motif was found in 12 TrnC homologs; *B. thuringiensis serovar pulsiensis* BGSC 4CC1 Bthur0012_58010, *Thermoanaerobacter mathranii* A3 Tmath_1971 and *Clostridium difficile* QCD-63q42 CdifQCD-6_020200015426 did not contain the motif. Tmath_1971 and CdifQCD-6_020200015426 were also lacking the GGE/DP and CX2CX5CX4C motif. Bthur0012_58010 did contain the GGEPLL and the CX2CX5CX4C motif. Other motifs of note include a SXDG motif located starting at alignment position 340 and conserved in 89% of the strains. A CEK/R and a MX_2_D/E motif were conserved in 68.75% of the proteins. Highly conserved single residues included, again, a number of highly conserved aspartic acid residues. One of four conserved aspartic acid residues was found to be adjacent to a glycine in TrnC and 7 other homologs. These are likely to be important for the maintenance of the SAM binding site [50].

**Table 1:** Highly conserved residues shared by trnD and 10 homologs.

From Alignment	Alignment position	% Conservation
**Motifs**		
YD	25–26	55
DE	70–71	55
CX_3_CX_2_C	123–131	100
GGXPLL	184–189	91
TNG	218–220	64
CX_2_CX_5_C	483–492	100
PC	420–421	64
**Single Residues**		
Y	25; 85	64; 73
G	82; 144; 425; 435; 450	82; 73; 82; 82; 64
D	192; 340; 393; 434	73; 73; 64; 73
K	195; 273; 309; 477	73; 64; 64; 64
F	231; 257	82; 73
N	235; 271	73; 64
I	237	73
S	264	64
P	382; 439	82; 73
C	440; 495;530	82; 73; 64

Residues are numbered according to their position in the multiple alignments.

**Table 2:** Highly conserved residues shared by trnC and 18 homologs.

From Alignment	Alignment position	% Conservation
**Motifs**		
CX_3_CX_2_C	202–209	89
GGEPLL	280–287	68
TTN	315–317	89
LL	320–321	63
SXDG	340–343	89
GXG	358–361	58
DG	547–548	63
PC	552–553	63
CE	553–555	68
GN	565–566	58
CX_2_CX_5_CX_4_C	597–610	100
CWA	600–602	53
**Single Residues**		
D	44;243;349;401	58; 95; 79; 74
G	137;538;566; 573	58; 84; 100; 84
T	199	74
Y	222; 281	63; 68
M	233	89
S	254	68
F	280; 289	84; 74
K	293; 300	100; 58
N	333	74
E	324; 346; 650	74; 74; 63
R	352	100
C	535; 553; 629	100; 100; 100

Residues are numbered according to their position in the multiple alignments.

**Figure 4:**
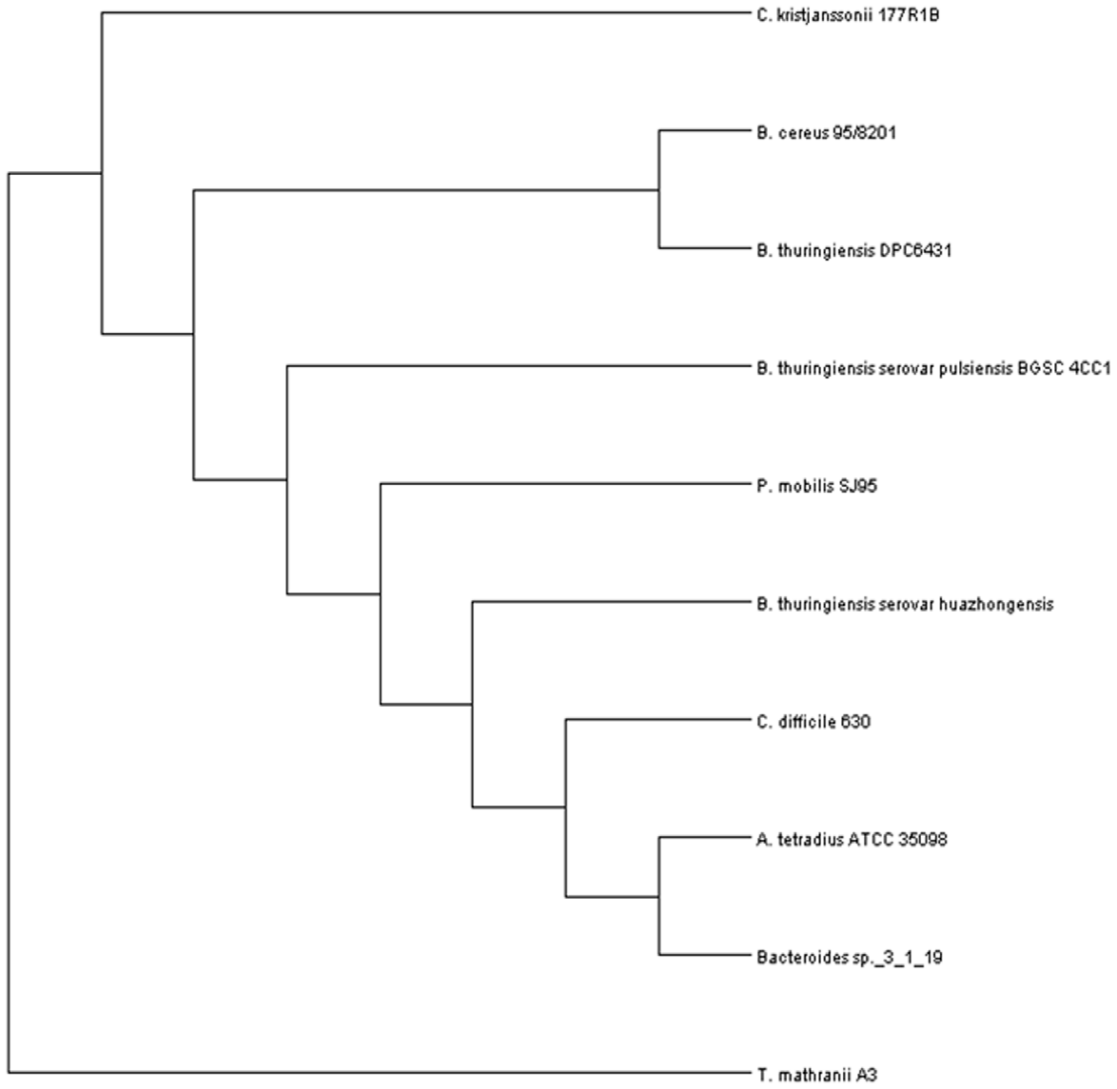
Rectangular cladogram of TrnD and all the TrnD homologs encountered during the screen. Cladogram was generated using the R package and visualised in DENDROSCOPE.

**Figure 5:**
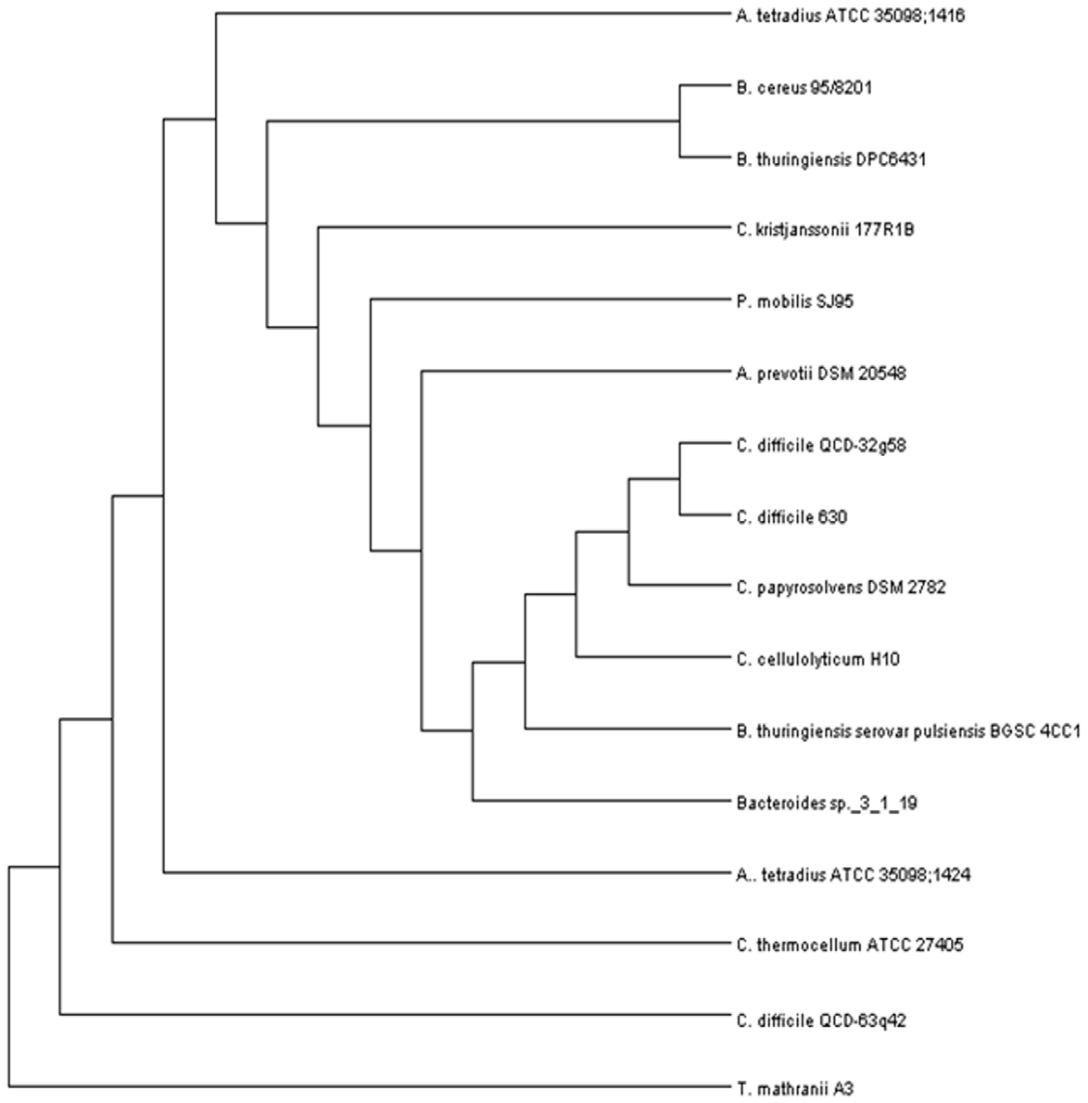
Rectangular cladogram of TrnC and all the TrnC homologs encountered during the screen. Cladogram was generated using the R package and visualised in DENDROSCOPE.

The conserved nature of these TrnC and Trn D homologs facilitated a phylogenetic analysis of their relatedness. A neighbour-joining tree generated from aligned conserved regions on the basis of percent identity was constructed for both sets of homologs. The unrooted cladogram of the Trn D homologs showed that *B. thuringiensis* DPC 6431 TrnD is most closely related to its homolog in *B. cereus* 95/8201, clustering together in the same branch (Fig. 4) This supports the findings from the in-silico screen. The cladogram of Trn C homologs (Fig. 5) is quite similar to that of the Trn D homologs. Once again *B. thuringiensis* DPC 6431 is shown to be most closely related to *B. cereus* 95/8201.

## Discussion

Thuricin CD, a bacteriocin with a narrow spectrum of activity which includes *C. difficile*, was recently identified as providing a potential therapeutic solution to this important pathogen [4]. *C. difficile* infection (CDI) is the leading cause of nosocomial infections associated with antibiotic use and is responsible for substantial morbidity and mortality world-wide [51]. The posttranslational modifications found in thuricin CD are unusual, and had not previously been associated with a two–peptide bacteriocin. In addition to potentially providing a treatment for CDAD, it, together with subtilosin A, may also represent a previously undefined structural class of bacteriocins, the sactibiotics. Bearing this in mind an *in-silico* approach was used to identify other thuricin CD-like gene clusters. *An in-silico approach* to identify strains that may be capable of bacteriocin production has recently become a viable alternative to culture based approaches as a consequence of the increasing generation and availability of genomic and metagenomic sequence data. While *in silico* identification of gene clusters in a strain will not always be lead to the detection of an associated bacteriocin, previous studies have shown that there is a high correlation [13], [52].

The initial *in silico* strategy adopted here resulted in the initial identification of 99 TrnC and 53 TrnD homologs. Further analysis led to the identification of 15 novel clusters which prior to this study had not been identified as potential bacteriocin-like clusters. These gene clusters are predominantly within the Firmicutes, with all but two gene clusters from species within this phylum. It was interesting to also find gene clusters within the Bacteroidetes phylum, namely *Bacteriodes* sp 3_1_19, and also within the Thermatogae phylum, namely *Petrotoga mobilis* SJ95, because bacteriocin production within these phyla is quite rare. The availability in the future of additional species corresponding to these genera will reveal whether these gene clusters are exceptional or not. The analysis of metagenomic sequence data further highlighted the distribution of gene clusters containing TrnC and TrnD-like genes. While at present it is not possible to predict definitively if these metagenomic homologues are encoded with bacteriocin-like gene clusters, the possibility that at least a portion of these encode novel sactibiotics is a fascinating prospect. Indeed, the harnessing of such novel antimicrobials from genomic/metagenomic material through heterologous expression or through isolation and investigation of corresponding natural producers should greatly expand the collection of sactibiotics at our disposal.

The importance of thuricin CD as a potential therapeutic against pathogenic *C.difficile* has previously been reported [4]; locating thuricin CD –like bacteriocins may have huge benefits to the field. Genomic mining with TrnC and TrnD from the Thuricin CD operon revealed 100 and 54 homologs respectively; resulting in the identification of 15 with novel potential sactibiotic-encoding gene clusters. The identification of additional clusters from diverse metagenomic samples highlights the broad distribution of potential sactibiotic gene clusters.

## Materials and Methods

### Sequence Analysis

The protein sequences of TrnC and TrnD from *Bacillus thuringiensis* DPC 6431 were used as driver sequences to mine bacterial genomes using the BLASTP web server on NCBI (www.ncbi.nlm.nih/BLAST) using default parameters [53]. Homology was defined as an e-value greater than 1e^-6^ and greater than 20% identity. Genes surrounding the TrnC and TrnD homologs were examined using the genome viewer on NCBI and also using the DNA sequence viewer ARTEMIS [54].

### Phylogenetic analysis

Protein alignments were generated by MUSCLE [55]. A phylogenetic tree was constructed using the TrnC and TrnD homologs identified in the screen. Protein trees were built using the R statistical package (http://cran.r-project.org *)* and the resultant phylogenetic trees were visualised using the DENDROSCOPE package [56].

### Metagenomic Analysis

The TrnC and TrnD protein sequences were used as driver sequences to search for homologs in metagenomic datasets. The metagenomic portal CAMERA (http://web.camera.calit2.net) [44] was used to BLAST against all publicly available metagenomic datasets. The criterion for homolog detection was a threshold of 1e^-6^ and greater than 20% identity.

## Supporting Information

Table S1 Bacterial genomes in which TrnC homologs were identified.
(DOC)

Table S2 Bacterial genomes in which TrnD homologs were identified.
(DOC)

Table S3 TrnC homologs in metagenomic databases.
(DOC)

Table S4 TrnD homologs in metagenomic databases.
(DOC)
