# Supplementary material for: Genome Mining for Radical SAM Protein Determinants Reveals Multiple Sactibiotic-Like Gene Clusters
Source: PLoS One. 2011 Jul 8;6(7):e20852. doi: 10.1371/journal.pone.0020852 (PMC3132745; doi:10.1371/journal.pone.0020852)
Supplement: Table S1 — Bacterial genomes in which TrnC homologs were identified. (DOC) [file pone.0020852.s001.doc]

**Suppl. Table 1.** Bacterial genomes in which TrnC homologs were identified.

|  | | Strain | Homolog | | | GenBank Accession no | | Identity (%) | | Similarity (%) | | E value |
| --- | --- | --- | --- | --- | --- | --- | --- | --- | --- | --- | --- | --- |
|  | Bacillus cereus 95/8201 | | | Radical SAM domain protein (bcere0016_53380) | ZP_04254230 | | 100 | | 100 | | 0 | |
|  | Caldicellulosiruptor kristjanssonii 177R1B | | | Radical SAM domain protein (Calkr_0222) | YP_004025400 | | 36 | | 57 | | 5.00E-77 | |
|  | Dictyoglomus thermophilum H-6-12 | | | Putative regulatory protein (DICTH_0218) | YP_002250101 | | 33 | | 52 | | 6.00E-049 | |
|  | | Petrotoga mobilis SJ95 | Radical SAM domain-containing protein (Pmob_0687) | | | YP_001567736 | | 33 | | 51 | | 1.00E-043 |
|  | | Geobacillus sp. Y412MC52 | Radical SAM domain protein (GYMC52DRAFT_2721) | | | ZP_04393571 | | 31 | | 53 | | 5.00E-40 |
|  | | Anaerococcus tetradius ATCC 35098 | Radical SAM-family protein (HMPREF0077_0726) | | | ZP_03930091 | | 28 | | 47 | | 7.00E-37 |
|  | | Anaerococcus prevotii DSM 20548 | Radical SAM domain protein (Apre_1837) | | | YP_003142411 | | 28 | | 46 | | 8.00E-37 |
|  | | Petrotoga mobilis SJ95 | Radical SAM domain protein (Pmob_0678) | | | YP_001567730 | | 29 | | 48 | | 3.00E-36 |
|  | | Clostridium botulinum A2 str. Kyoto | Radical SAM domain protein (CLM_3252) | | | YP_002805381 | | 30 | | 48 | | 2.00E-33 |
|  | | Clostridium sporogenes ATCC 15579 | Hypothetical protein CLOSPO_01331 | | | ZP_02994212 | | 29 | | 47 | | 9.00E-33 |
|  | | Paenibacillus larvae subsp. larvae BRL-230010 | Radical SAM domain protein (Plarl_010100003615) | | | ZP_02326726 | | 38 | | 55 | | 2.00E-32 |
|  | | Clostridium botulinum NCTC 2916 | Radical SAM-family protein (CBN_2847) | | | ZP_02613955 | | 28 | | 46 | | 2.00E-31 |
|  | | Clostridium papyrosolvens DSM 2782 | Radical SAM domain protein (CpapDRAFT_0560) | | | ZP_05494390 | | 28 | | 47 | | 3.00E-30 |
|  | | Clostridium difficile QCD-32g58 | Hypothetical protein CdifQ_04000180 | | | ZP_01805077 | | 25 | | 46 | | 5.00E-30 |
|  | | Clostridium difficile 630 | Radical SAM-family protein (CD0162) | | | YP_001086629 | | 25 | | 46 | | 5.00E-30 |
|  | | B. thuringiensis serovar pulsiensis BGSC 4CC1 | Radical SAM domain protein (bthur0012_58010) | | | ZP_04082097 | | 30 | | 49 | | 5.00E-30 |
|  | | Clostridium cellulolyticum H10 | Radical SAM domain protein (Ccel_0328) | | | YP_002504695 | | 27 | | 47 | | 7.00E-30 |
|  | | Clostridium botulinum B1 str. Okra | Radical SAM domain-containing protein (CLD_1367) | | | YP_001782803 | | 28 | | 47 | | 9.00E-30 |
|  | | Ruminococcus obeum ATCC 29174 | Hypothetical protein RUMOBE_00367 | | | ZP_01962654 | | 29 | | 47 | | 1.00E-29 |
|  | | Clostridium botulinum F str. Langeland | Radical SAM domain protein (CLI_3234) | | | YP_001392446 | | 28 | | 46 | | 2.00E-29 |
|  | | Clostridium cellulolyticum H10 | Radical SAM domain protein (Ccel_2370) | | | YP_002506683.1 | | 27 | | 46 | | 2.00E-29 |
|  | | Ruminococcus sp. SR1/5 | Arylsulfatase regulator (Fe-S oxidoreductase) (CK1_23720) | | | CBL20339 | | 27 | | 45 | | 3.00E-29 |
|  | | Clostridium papyrosolvens DSM 2782 | Radical SAM domain protein (CpapDRAFT_1026) | | | ZP_05494856 | | 28 | | 47 | | 3.00E-28 |
|  | | Desulfovibrio magneticus RS-1 | Hypothetical protein (DMR_32270) | | | YP_002954604 | | 26 | | 46 | | 4.00E-28 |
|  | | Clostridium papyrosolvens DSM 2782 | Radical SAM domain protein (CpapDRAFT_0066) | | | ZP_05493896 | | 26 | | 49 | | 4.00E-28 |
|  | | Clostridium acetobutylicum ATCC 824 | Fe-S oxidoreductase (CA_C0658) | | | NP_347295 | | 27 | | 44 | | 8.00E-27 |
|  | | Clostridium cellulolyticum H10 | Radical SAM domain protein (Ccel_2600) | | | YP_002506908 | | 26 | | 49 | | 2.00E-26 |
|  | | Anaerococcus vaginalis ATCC 51170 | Fe-S oxidoreductase (HMPREF0078_0423) | | | ZP_05472166 | | 27 | | 44 | | 3.00E-26 |
|  | | Anaerococcus hydrogenalis DSM 7454 | Hypothetical protein (ANHYDRO_00108) | | | ZP_03303719 | | 27 | | 45 | | 2.00E-25 |
|  | | Clostridium spiroforme DSM 1552 | Hypothetical protein (CLOSPI_01156) | | | ZP_02867326 | | 28 | | 45 | | 3.00E-24 |
|  | | Eubacterium rectale M104/1 | Arylsulfatase regulator (Fe-S oxidoreductase) (ERE_01150) | | | CBK92260 | | 24 | | 43 | | 2.00E-23 |
|  | | Roseburia intestinalis L1-82 | Putative Fe-S oxidoreductase (ROSINTL182_09433) | | | ZP_04745996 | | 24 | | 45 | | 2.00E-23 |
|  | | Anaerococcus tetradius ATCC 35098 | Radical SAM domain protein (HMPREF0077_1424) | | | ZP_03930789 | | 29 | | 48 | | 3.00E-23 |
|  | | Bacteroides fragilis NCTC 9343 | Regulatory protein (BF4197) | | | YP_213762 | | 26 | | 45 | | 3.00E-23 |
|  | | Clostridium nexile DSM 1787 | Hypothetical protein (CLONEX_03364) | | | ZP_03291143 | | 26 | | 46 | | 3.00E-23 |
|  | | Clostridium phytofermentans ISDg | Radical SAM domain-containing protein (Cphy_2677) | | | YP_001559777 | | 26 | | 42 | | 8.00E-23 |
|  | | Clostridium butyricum 5521 | Radical SAM-family protein (CBY_1639) | | | ZP_02950343 | | 28 | | 43 | | 1.00E-22 |
|  | | Clostridium difficile QCD-63q42 | Radical SAM-family protein (CdifQCD-6_020200015446) | | | ZP_05331187 | | 26 | | 46 | | 3.00E-21 |
|  | | Bacteroides sp. 3_2_5 | Conserved hypothetical protein (BSHG_04191) | | | ZP_04844671 | | 25 | | 43 | | 6.00E-21 |
|  | | Bacteroides uniformis ATCC 8492 | Hypothetical protein (BACUNI_03551) | | | ZP_02072107 | | 26 | | 44 | | 6.00E-21 |
|  | | Parabacteroides distasonis ATCC 8503 | Putative Fe-S oxidoreductase (BDI_3163) | | | YP_001304491 | | 25 | | 43 | | 8.00E-21 |
|  | | Bacteroides sp. D20 | Conserved hypothetical protein (HMPREF0969_00927) | | | ZP_06200607 | | 27 | | 45 | | 9.00E-21 |
|  | | Bacteroides sp. 2_1_7 | Putative Fe-S oxidoreductase (B2_010100003588) | | | ZP_05285086 | | 25 | | 43 | | 1.00E-20 |
|  | | Bacteroides sp. 2_1_16 | Conserved hypothetical protein (HMPREF0101_04349) | | | ZP_06095349 | | 25 | | 43 | | 1.00E-20 |
|  | | Arcobacter butzleri RM4018 | Quinohemoprotein amine dehydrogenase (Abu_0466) | | | YP_001489410 | | 28 | | 46 | | 3.00E-20 |
|  | | Clostridium carboxidivorans P7 | Radical SAM domain protein (CcarbDRAFT_3571) | | | ZP_05393571 | | 31 | | 50 | | 4.00E-20 |
|  | | Clostridium acetobutylicum ATCC 824 | Heme biosynthesis (nirJ-2) family protein (CA_C2279) | | | NP_348896 | | 30 | | 48 | | 5.00E-20 |
|  | | Clostridium botulinum Bf | Radical SAM-family protein (CBB_2308) | | | ZP_02619747 | | 30 | | 53 | | 6.00E-20 |
|  | | Clostridium botulinum A3 str. Loch Maree | Radical SAM domain-containing protein (CLK_2457) | | | YP_001788384 | | 25 | | 40 | | 1.00E-19 |
|  | | Bacteroides fragilis 3_1_12 | Putative dehydrogenase (Bfra3_010100021545) | | | ZP_05283865 | | 24 | | 44 | | 1.00E-19 |
|  | | Clostridium botulinum F str. Langeland | Radical SAM domain protein (CLI_3123) | | | YP_001392342 | | 29 | | 45 | | 2.00E-19 |
|  | | Thermodesulfovibrio yellowstonii DSM 11347 | Heme biosynthesis (NirJ-2) family protein, putative (THEYE_A1288) | | | YP_002249102 | | 35 | | 52 | | 2.00E-19 |
|  | | Pelotomaculum thermopropionicum SI | Arylsulfatase regulator (PTH_0885) | | | YP_001211435 | | 28 | | 41 | | 7.00E-19 |
|  | | Paracoccus denitrificans | Quinohemoprotein amine dehydrogenase unknown subunit | | | BAB78727 | | 26 | | 42 | | 8.00E-19 |
|  | | Clostridium perfringens SM101 | Radical SAM domain-containing protein (CPR_1909) | | | YP_699219 | | 30 | | 49 | | 1.00E-18 |
|  | | Clostridium perfringens E str. JGS1987 | Radical SAM domain protein (AC3_2475) | | | ZP_02630879 | | 30 | | 49 | | 1.00E-18 |
|  | | Clostridium perfringens str. 13 | AstB/chuR-related protein (CPE1943) | | | NP_562859 | | 39 | | 49 | | 1.00E-18 |
|  | | Clostridium perfringens ATCC 13124 | Radical SAM domain-containing protein (CPF_2198) | | | YP_696622 | | 30 | | 49 | | 1.00E-18 |
|  | | Clostridium cellulolyticum H10 | Radical SAM domain protein (Ccel_0534) | | | YP_002504896 | | 29 | | 46 | | 2.00E-18 |
|  | | Paracoccus denitrificans PD1222 | Radical SAM domain protein (Pden_1704) | | | YP_915497 | | 25 | | 42 | | 3.00E-18 |
|  | | Clostridium butyricum 5521 | Heme biosynthesis (CBY_3903) | | | ZP_02950569 | | 31 | | 51 | | 3.00E-18 |
|  | | Thermoanaerobacter mathranii A3 | Radical SAM domain protein (Tmath_1071) | | | YP_003676802 | | 29 | | 47 | | 3.00E-18 |
|  | | Thermoanaerobacter ethanolicus CCSD1 | Radical SAM domain protein (TeCCSD1DRAFT_1975) | | | ZP_05493437 | | 28 | | 47 | | 3.00E-18 |
|  | | Clostridium beijerinckii NCIMB 8052 | Radical SAM domain protein (Cbei_4157) | | | YP_001311223 | | 30 | | 49 | | 3.00E-18 |
|  | | Ruminococcus gnavus ATCC 29149 | Hypothetical protein (RUMGNA_01458) | | | ZP_02040694 | | 32 | | 54 | | 4.00E-18 |
|  | | Thermoanaerobacter sp. X514 | Radical SAM domain-containing protein (Teth514_1460) | | | YP_001663085 | | 28 | | 47 | | 5.00E-18 |
|  | | Clostridium hiranonis DSM 13275 | Hypothetical protein (CLOHIR_00257) | | | ZP_03292314 | | 34 | | 60 | | 5.00E-18 |
|  | | Clostridium botulinum Bf | Radical SAM domain protein (CBB_3359) | | | ZP_02616418 | | 29 | | 45 | | 5.00E-18 |
|  | | Clostridium botulinum NCTC 2916 | Radical SAM domain protein (CBN_3110) | | | ZP_02614222 | | 29 | | 45 | | 5.00E-18 |
|  | | Ruminococcus sp.SR1/5 | Arylsulfatase regulator (Fe-S oxidoreductase) (CK1_32180) | | | CBL21030 | | 34 | | 52 | | 5.00E-18 |
|  | | Clostridium hiranonis DSM 13275 | Hypothetical protein (CLOHIR_02000) | | | ZP_03294049 | | 25 | | 48 | | 6.00E-18 |
|  | | Clostridium papyrosolvens DSM 2782 | Radical SAM domain protein (CpapDRAFT_1563) | | | ZP_05495393 | | 28 | | 48 | | 6.00E-18 |
|  | | Carboxydibrachium pacificum DSM 12653 | Radical SAM domain protein (CDSM653_53) | | | ZP_05091427 | | 35 | | 54 | | 6.00E-18 |
|  | | Clostridium difficile QCD-32g58 | Hypothetical protein (CdifQ_04003206) | | | ZP_01802234 | | 27 | | 47 | | 7.00E-18 |
|  | | Clostridium difficile 630 | Putative radical SAM family protein (CD2749) | | | YP_001089261 | | 27 | | 47 | | 7.00E-18 |
|  | | Clostridium sp. 7_2_43FAA | Radical SAM domain-containing protein (CSBG_01102) | | | ZP_05130582 | | 30 | | 48 | | 2.00E-17 |
|  | | Bacteroides sp. 3_1_19 | Dehydrogenase (HMPREF0104_00298) | | | ZP_06984105 | | 23 | | 44 | | 2.00E-17 |
|  | | Oribacterium sinus F0268 | Radical SAM domain protein (HMPREF6123_0410) | | | ZP_03990471 | | 29 | | 48 | | 2.00E-17 |
|  | | Clostridium difficile QCD-23m63 | Putative radical SAM family protein (CdifQCD-2_020200013816) | | | ZP_05402143 | | 27 | | 46 | | 3.00E-17 |
|  | | Thermoanaerobacter pseudethanolicus | Radical SAM domain-containing protein (Teth39_1021) | | | YP_001665012 | | 28 | | 47 | | 3.00E-17 |
|  | | Anaerococcus tetradius ATCC 35098 | Radical SAM domain protein (HMPREF0077_1416) | | | ZP_03930781 | | 25 | | 42 | | 3.00E-17 |
|  | | Pseudomonas fluorescens Pf0-1 | Radical SAM family protein (Pfl01_2974) | | | YP_348705 | | 25 | | 41 | | 3.00E-17 |
|  | | Dethiobacter alkaliphilus AHT 1 | Radical SAM domain protein (DealDRAFT_0434) | | | ZP_03728579 | | 28 | | 44 | | 3.00E-17 |
|  | | Anaerococcus lactolyticus ATCC 51172 | Radical SAM domain protein (HMPREF0072_2181) | | | ZP_03917094 | | 29 | | 45 | | 4.00E-17 |
|  | | Clostridium botulinum A str. ATCC 3502 | Radical SAM domain protein (CBO3064) | | | YP_001255556 | | 28 | | 44 | | 5.00E-17 |
|  | | Bacteroides sp. 3_2_5 | Regulatory protein (BSHG_03481) | | | ZP_04843961 | | 22 | | 42 | | 5.00E-17 |
|  | | Shuttleworthia satelles DSM 14600 | Hypothetical protein (GCWU000342_00730) | | | ZP_04454733 | | 25 | | 48 | | 6.00E-17 |
|  | | Clostridium sporogenes ATCC 15579 | Hypothetical protein (CLOSPO_01947) | | | ZP_02994828 | | 28 | | 44 | | 6.00E-17 |
|  | | Anaerococcus prevotii DSM 20548 | Radical SAM domain protein (Apre_1008) | | | YP_003152757 | | 29 | | 45 | | 6.00E-17 |
|  | | Clostridium lentocellum DSM 5427 | Radical SAM domain protein (CloleDRAFT_0460) | | | ZP_06882374 | | 27 | | 48 | | 7.00E-17 |
|  | | Bacteroides fragilis 3_1_12 | Regulatory protein (Bfra3_010100020285) | | | ZP_05283620 | | 22 | | 42 | | 8.00E-17 |
|  | | Clostridium thermocellum JW20 | Radical SAM domain protein (Cther_2305) | | | ZP_06248646 | | 28 | | 46 | | 9.00E-17 |
|  | | Clostridium botulinum B1 str. Okra | Radical SAM domain-containing protein (CLD_1476) | | | YP_001782699 | | 31 | | 48 | | 1.00E-16 |
|  | | Clostridium tetani E88 | Transcriptional regulatory protein (CTC02206) | | | NP_782755 | | 28 | | 43 | | 2.00E-16 |
|  | | Citreicella sp. SE45 | Radical SAM domain protein | | | ZP_05779920 | | 24 | | 42 | | 2.00E-16 |
|  | | Pelotomaculum thermopropionicum SI | Fe-S oxidoreductases | | | YP_001213413 | | 25 | | 44 | | 2.00E-16 |
|  | | Thermincola sp. JR | Radical SAM domain protein | | | YP_003641684 | | 26 | | 43 | | 3.00E-16 |
|  | | Anaerococcus tetradius ATCC 35098 | Radical SAM domain protein HMPREF0077_0209 | | | ZP_03929574 | | 29 | | 44 | | 3.00E-16 |
|  | | Clostridium thermocellum ATCC 27405 | Radical SAM family protein | | | YP_001037333 | | 27 | | 46 | | 3.00E-16 |
|  | | Alkaliphilus metalliredigens QYMF | Radical SAM domain-containing protein | | | YP_001320159 | | 25 | | 45 | | 3.00E-016 |
