# Supplementary material for: Genome Mining for Radical SAM Protein Determinants Reveals Multiple Sactibiotic-Like Gene Clusters
Source: PLoS One. 2011 Jul 8;6(7):e20852. doi: 10.1371/journal.pone.0020852 (PMC3132745; doi:10.1371/journal.pone.0020852)
Supplement: Table S2 — Bacterial genomes in which TrnD homologs were identified. (DOC) [file pone.0020852.s002.doc]

**Suppl. Table 2.** Bacterial genomes in which TrnD homologs were identified.

| Strain | Homolog | GenBank Accession No | Identity (%) | Similarity (%) | E value |
| --- | --- | --- | --- | --- | --- |
| Bacillus cereus 95/8201 | Radical SAM domain protein (bcere0016_53390) | ZP_04254231.1| | 99 | 100 | 0 |
| Caldicellulosiruptor kristjanssonii 177R1B | Radical SAM domain protein (Calkr_0223) | YP_004025401 | 32 | 56 | 7.00E-61 |
| Thermosinus carboxydivorans Nor1 draft | Radical SAM domain protein (TcarDRAFT_1734) | ZP_01665992.1| | 23 | 41 | 4.00E-26 |
| Bacillus thuringiensis serovar huazhongensis BGSC 4BD1 draft | hypothetical protein bthur0011_60830 | ZP_04088321.1| | 25 | 44 | 2.00E-23 |
| Bacillus thuringiensis serovar pulsiensis BGSC 4CC1 draft | hypothetical protein bthur0012_58000 | ZP_04082096.1| | 25 | 44 | 8.00E-23 |
| Petrotoga mobilis SJ95 | hypothetical protein Pmob_0677 | YP_001567729.1| | 23 | 42 | 1.00E-18 |
| Geobacillus sp. Y412MC52 draft | Radical SAM domain protein (GYMC52DRAFT_2720) | ZP_04393570.1| | 24 | 43 | 2.00E-17 |
| Thermococcus sp. AM4 draft | conserved hypothetical protein (TAM4_1119) | ZP_04879806.1| | 27 | 50 | 3.00E-14 |
| Pyrococcus abyssi GE5 | hypothetical protein PAB0111 | NP_125861.1| | 34 | 59 | 1.00E-12 |
| Thermococcus gammatolerans EJ3 | hypothetical protein TGAM_0171 | YP_002958537.1| | 27 | 55 | 1.00E-12 |
| Thermococcus barophilus MP draft | hypothetical protein TERMP_1340 | ZP_04876632.1| | 29 | 54 | 2.00E-12 |
| Thermococcus kodakarensis KOD1 | hypothetical protein TK2114 | YP_184527.1| | 29 | 55 | 3.00E-12 |
| Pyrococcus horikoshii OT3 | hypothetical protein PH0185 | NP_142184.1| | 35 | 61 | 7.00E-12 |
| Pyrococcus furiosus DSM 3638 | hypothetical protein PF0101 | NP_577830.1| | 23 | 47 | 1.00E-11 |
| Thermococcus onnurineus NA1 | hypothetical protein TON_1619 | YP_002308006.1| | 25 | 48 | 3.00E-11 |
| Thermococcus sibiricus MM 739 | Predicted Fe-S oxidoreductase (TSIB_1857) | YP_002995255.1| | 24 | 52 | 8.00E-10 |
| Clostridium difficile 630 | radical SAM-family protein (CD0162) | YP_001086629.1| | 24 | 47 | 2.00E-08 |
| Clostridium difficile QCD-32g58 draft | hypothetical protein CdifQ_04000180 | ZP_01805077.1| | 24 | 47 | 4.00E-08 |
| Thermosipho melanesiensis BI429 | Radical SAM domain protein (Tmel_0409) | YP_001305663.1| | 25 | 52 | 8.00E-08 |
| Kosmotoga olearia TBF 19.5.1 | Radical SAM domain protein (Kole_0770) | YP_002940486.1| | 28 | 46 | 2.00E-07 |
| Thermococcus kodakarensis KOD1 | hypothetical protein TK0675 | YP_183088.1| | 24 | 43 | 2.00E-07 |
| Clostridium papyrosolvens DSM 2782 draft | Radical SAM domain protein (CpapDRAFT_0560) | ZP_05494390.1| | 30 | 49 | 2.00E-07 |
| Clostridium botulinum A2 str. Kyoto | radical SAM domain protein (CLM_3252) | YP_002805381.1| | 24 | 46 | 2.00E-07 |
| Clostridium botulinum NCTC 2916 draft | radical SAM-family protein (CBN_2847) | ZP_02613955.1| | 25 | 45 | 2.00E-07 |
| Thermincola sp. JR | Radical SAM domain protein (TherJR_2953) | YP_003641684.1| | 22 | 41 | 2.00E-07 |
| Bacteroides vulgatus ATCC 8482 | putative Fe-S oxidoreductase (BVU_3750) | YP_001300983.1| | 25 | 44 | 3.00E-07 |
| Desulfitobacterium hafniense Y51 | hypothetical protein DSY5020 | YP_521253.1| | 25 | 45 | 4.00E-07 |
| Anaerococcus prevotii DSM 20548 | Radical SAM domain protein (Apre_1754) | YP_003142336.1| | 29 | 46 | 5.00E-07 |
| Chlorobium phaeobacteroides BS1 | Radical SAM domain protein (Cphamn1_0679) | YP_001959118.1| | 30 | 49 | 6.00E-07 |
| uncultured archaeon n/a | conserved hypothetical protein, radical SAM superfamily(BSM_01840) | CBH36707.1| | 21 | 37 | 6.00E-07 |
| Dictyoglomus thermophilum H-6-12 | hypothetical protein DICTH_0216 | YP_002250099.1| | 27 | 48 | 7.00E-07 |
| Thermoanaerobacterium thermosaccharolyticum DSM 571 draft | Radical SAM domain protein (TtheDRAFT_2448) | ZP_05337222.1| | 23 | 43 | 7.00E-07 |
| Clostridium acetobutylicum ATCC 824 | Heme biosynthesis (nirJ-2) family protein (CA_C2279) | NP_348896.1| | 22 | 42 | 8.00E-07 |
| Alkaliphilus oremlandii OhILAs | radical SAM domain-containing protein (Clos_1718) | YP_001513254.1| | 21 | 42 | 9.00E-07 |
| Natranaerobius thermophilus JW/NM-WN-LF | molybdenum cofactor biosynthesis protein A (Nther_1265) | YP_001917436.1| | 25 | 44 | 1.00E-06 |
| Clostridium cellulolyticum H10 | Radical SAM domain protein (Ccel_0328) | YP_002504695.1| | 26 | 47 | 2.00E-06 |
| Thermoanaerobacter ethanolicus CCSD1 draft | Radical SAM domain protein (TeCCSD1DRAFT_0583) | ZP_05492046.1| | 25 | 40 | 2.00E-06 |
| Caldicellulosiruptor saccharolyticus DSM 8903 | radical SAM domain-containing protein (Csac_0977) | YP_001179782.1| | 31 | 50 | 2.00E-06 |
| Serratia odorifera 4Rx13 draft | molybdenum cofactor biosynthesis protein A (SOD_i00390) | ZP_06192887.1| | 29 | 45 | 2.00E-06 |
| Clostridium botulinum B1 str. Okra | radical SAM domain-containing protein (CLD_1367) | YP_001782803.1| | 24 | 42 | 2.00E-06 |
| Prosthecochloris aestuarii DSM 271 | Radical SAM domain protein (Paes_2372) | YP_002019717.1| | 25 | 41 | 2.00E-06 |
| Clostridium botulinum F str. Langeland | radical SAM domain-containing protein (CLI_3234) | YP_001392446.1| | 24 | 42 | 3.00E-06 |
| Fusobacterium sp. D12 draft | radical SAM domain-containing protein (FuD12_010100000792) | ZP_05626781.1| | 22 | 37 | 3.00E-06 |
| Clostridium tetani E88 | transcriptional regulatory protein (CTC02206) | NP_782755.1| | 23 | 44 | 3.00E-06 |
| Thermoanaerobacter mathranii subsp. mathranii str. A3 | Radical SAM domain protein (Tmath_1976) | YP_003677673.1| | 30 | 46 | 3.00E-06 |
| Anaerofustis stercorihominis DSM 17244 draft | hypothetical protein ANASTE_02092 | ZP_02862865.1| | 28 | 47 | 4.00E-06 |
| Clostridium papyrosolvens DSM 2782 draft | Radical SAM domain protein (CpapDRAFT_1563) | ZP_05495393.1| | 22 | 41 | 4.00E-06 |
| Escherichia albertii TW07627 draft | molybdenum cofactor biosynthesis protein A (ESCAB7627_3633) | ZP_02903554.1| | 27 | 45 | 4.00E-06 |
| Bacillus cereus 95/8201 draft | Radical SAM domain protein (bcere0016_53380) | ZP_04254230.1| | 22 | 48 | 5.00E-06 |
| Candidatus Korarchaeum cryptofilum OPF8 | radical SAM domain-containing protein (Kcr_1025) | YP_001737454.1| | 33 | 53 | 5.00E-06 |
| Clostridium hiranonis DSM 13275 draft | hypothetical protein CLOHIR_00257 | ZP_03292314.1| | 22 | 42 | 6.00E-06 |
| uncultured archaeon n/a | conserved hypothetical protein radical SAM superfamily (BSM_25980) | CBH39120.1| | 22 | 40 | 7.00E-06 |
| Thermotoga lettingae TMO | radical SAM domain-containing protein (Tlet_1232) | YP_001470856.1| | 26 | 44 | 8.00E-06 |
| Anaerocellum thermophilum DSM 6725 | Radical SAM domain protein (Athe_0623) | YP_002572520.1| | 25 | 44 | 9.00E-06 |
| Escherichia fergusonii ATCC 35469 | molybdenum cofactor biosynthesis protein A (EFER_2330) | YP_002383446.1| | 26 | 45 | 9.00E-06 |
