# Supplementary material for: Genome Mining for Radical SAM Protein Determinants Reveals Multiple Sactibiotic-Like Gene Clusters
Source: PLoS One. 2011 Jul 8;6(7):e20852. doi: 10.1371/journal.pone.0020852 (PMC3132745; doi:10.1371/journal.pone.0020852)
Supplement: Table S3 — TrnC homologs in metagenomic databases. (DOC) [file pone.0020852.s003.doc]

**Suppl Table 3 TrnC homologs in metagenomic databases**

| Protein function | Metagenome | Location | % identity | e-value |
| --- | --- | --- | --- | --- |
| **radical SAM domain protein** | **Open Ocean** | **Sargasso Sea, Bermuda** | **30** | **1.88e-32** |
| **radical SAM** | **Hypersaline Lagoons** | **Galapagos Islands, Ecuador** | **33** | **4.48e-30** |
| **radical SAM** | **Hypersaline Lagoons** | **Galapagos Islands, Ecuador** | **34** | **7.31e-30** |
| **Hypothetical Protein** | **Open Ocean** | **Sargasso Sea, Bermuda** | **25** | **8.67e-30** |
| **radical SAM domain protein** | **Open Ocean** | **Sargasso Sea, Bermuda** | **28** | **1.03e-29** |
| **radical SAM domain protein** | **Open Ocean** | **Sargasso Sea, Bermuda** | **29** | **6.11e-27** |
| **radical SAM domain protein** | **Open Ocean** | **Sargasso Sea, Bermuda** | **29** | **9.59e-27** |
| **radical SAM domain protein** | **Open Ocean** | **Sargasso Sea, Bermuda** | **27** | | **2.11e-26** |  | | --- | --- | |
| **radical SAM domain protein** | **Open Ocean** | **Sargasso Sea, Bermuda** | **28** | **3.06e-26** |
| **radical SAM domain protein** | **Mangrove** | **Isabella Island, Galapagos Islands, Ecuador** | **38** | **4.55e-26** |
| **radical SAM domain protein** | **Open Ocean** | **Sargasso Sea, Bermuda** | **29** | **8.68e-26** |
| **radical SAM domain protein** | **Open Ocean** | **Sargasso Sea, Bermuda** | **27** | **1.94e-25** |
| **radical SAM domain protein** | **Open Ocean** | **Sargasso Sea, Bermuda** | **28** | **6.87e-25** |
| **radical SAM domain protein** | **Open Ocean** | **Sargasso Sea, Bermuda** | **27** | **8.54e-25** |
| **radical SAM domain protein** | **Open Ocean** | **Sargasso Sea, Bermuda** | **28** | **1.81e-24** |
| **radical SAM domain protein** | **Open Ocean** | **Sargasso Sea, Bermuda** | **26** | **1.83e-24** |
| **Hypothetical Protein** | **Open Ocean** | **Sargasso Sea, Bermuda** | **29** | **2.76e-24** |
| **heme biosynthesis** | **Open Ocean** | **Caribbean Sea, Mexico** | **23** | **4.80e-24** |
| **radical SAM domain protein** | **Open Ocean** | **Sargasso Sea, Bermuda** | **30** | **4.96e-24** |
| **radical SAM domain protein** | **Open Ocean** | **Sargasso Sea, Bermuda** | **26** | **7.21e-24** |
| **radical SAM** | **Hypersaline Lagoons** | **Galapagos Islands, Ecuador** | **37** | **1.73e-23** |
| **radical SAM domain protein** | **Open Ocean** | **Sargasso Sea, Bermuda** | **28** | **1.73e-23** |
| **radical SAM domain protein** | **Open Ocean** | **Sargasso Sea, Bermuda** | **26** | **1.95e-22** |
| **radical SAM domain protein** | **Open Ocean** | **Sargasso Sea, Bermuda** | **29** | **4.57e-22** |
| **Hypothetical Protein** | **Open Ocean** | **Sargasso Sea, Bermuda** | **34** | **1.04e-21** |
| **radical SAM domain protein** | **Open Ocean** | **Sargasso Sea, Bermuda** | **27** | **1.04e-21** |
| **radical SAM domain protein** | **Mangrove** | **Isabella Island, Galapagos Islands, Ecuador** | **33** | **1.13e-21** |
| **radical SAM domain protein** | **Open Ocean** | **Sargasso Sea, Bermuda** | **26** | **1.50e-21** |
| **radical SAM** | **Hypersaline Lagoons** | **Galapagos Islands, Ecuador** | **33** | **2.90e-21** |
| **radical SAM domain protein** | **Open Ocean** | **Sargasso Sea, Bermuda** | **28** | **3.62e-21** |
| **radical SAM domain protein** | **Open Ocean** | **Sargasso Sea, Bermuda** | **29** | **3.90e-21** |
| **radical SAM domain protein** | **Open Ocean** | **Sargasso Sea, Bermuda** | **33** | **5.09e-21** |
| **radical SAM domain protein** | **Open Ocean** | **Sargasso Sea, Bermuda** | **33** | **5.58e-21** |
| **radical SAM domain protein** | **Open Ocean** | **Sargasso Sea, Bermuda** | **33** | **5.73e-21** |
| **radical SAM domain protein** | **Open Ocean** | **Sargasso Sea, Bermuda** | **33** | **6.12e-21** |
| **radical SAM domain protein** | **Open Ocean** | **Sargasso Sea, Bermuda** | **34** | **6.60e-21** |
| **radical SAM domain protein** | **Open Ocean** | **Sargasso Sea, Bermuda** | **32** | **7.05e-21** |
| **radical SAM domain protein** | **Open Ocean** | **Sargasso Sea, Bermuda** | **33** | **7.67e-21** |
| **radical SAM domain protein** | **Open Ocean** | **Sargasso Sea, Bermuda** | **33** | **8.47e-21** |
| **radical SAM domain protein** | **Open Ocean** | **Sargasso Sea, Bermuda** | **25** | **9.58e-21** |
| **radical SAM domain protein** | **Mangrove** | **Galapagos Islands, Ecuador** | **31** | **1.04e-20** |
| **radical SAM domain protein** | **Open Ocean** | **Sargasso Sea, Bermuda** | **25** | **1.42e-20** |
| **radical SAM domain protein** | **Open Ocean** | **Sargasso Sea, Bermuda** | **30** | **1.54e-20** |
| **radical SAM domain protein** | **Open Ocean** | **Sargasso Sea, Bermuda** | **29** | **1.82e-20** |
| **radical SAM domain protein** | **Open Ocean** | **Sargasso Sea, Bermuda** | **30** | **2.36e-20** |
| **radical SAM domain protein** | **Open Ocean** | **Sargasso Sea, Bermuda** | **29** | | **2.70e-20** |  | | --- | --- | |
| **Hypothetical Protein** | **Open Ocean** | **Sargasso Sea, Bermuda** | **33** | **3.99e-20** |
| **radical SAM domain protein** | **Open Ocean** | **Sargasso Sea, Bermuda** | **29** | **4.06e-20** |
| **radical SAM domain protein** | **Open Ocean** | **Sargasso Sea, Bermuda** | **31** | **8.74e-20** |
| **radical SAM domain protein** | **Open Ocean** | **Sargasso Sea, Bermuda** | **36** | **9.66e-20** |
| **radical SAM domain protein** | **Open Ocean** | **Sargasso Sea, Bermuda** | **36** | **1.02e-19** |
| **radical SAM domain protein** | **Open Ocean** | **Sargasso Sea, Bermuda** | **36** | **1.15e-19** |
| **radical SAM domain protein** | **Open Ocean** | **Sargasso Sea, Bermuda** | **31** | **1.26e-19** |
| **radical SAM domain protein** | **Open Ocean** | **Sargasso Sea, Bermuda** | **31** | **1.27e-19** |
| **radical SAM domain protein** | **Open Ocean** | **Sargasso Sea, Bermuda** | **36** | **1.30e-19** |
| **radical SAM domain protein** | **Open Ocean** | **Sargasso Sea, Bermuda** | **36** | **1.39e-19** |
| **radical SAM** | **Hypersaline Lagoons** | **Galapagos Islands, Ecuador** | **28** | **1.95e-19** |
| **radical SAM domain protein** | **Open Ocean** | **Sargasso Sea, Bermuda** | **25** | **5.76e-19** |
| **Hypothetical Protein** | **Open Ocean** | **Sargasso Sea, Bermuda** | **35** | **5.81e-19** |
| **radical SAM domain protein** | **Mangrove** | **Galapagos Islands, Ecuador** | **33** | **6.04e-19** |
| **radical SAM domain protein** | **Mangrove** | **Galapagos Islands, Ecuador** | **33** | **6.20e-19** |
| **radical SAM domain protein** | **Mangrove** | **Galapagos Islands, Ecuador** | **22** | **9.69e-19** |
| **radical SAM** | **Hypersaline Lagoons** | **Galapagos Islands, Ecuador** | **31** | **1.83e-18** |
| **radical SAM** | **Hypersaline Lagoons** | **Galapagos Islands, Ecuador** | **35** | **2.01e-18** |
| **radical SAM domain protein** | **Mangrove** | **Galapagos Islands, Ecuador** | **30** | **3.73e-18** |
| **radical SAM domain protein** | **Open Ocean** | **Sargasso Sea, Bermuda** | **25** | **3.77e-18** |
| **Hypothetical Protein** | **Open Ocean** | **Sargasso Sea, Bermuda** | **33** | **4.33e-18** |
| **Hypothetical Protein** | **Open Ocean** | **Sargasso Sea, Bermuda** | **33** | **4.71e-18** |
| **radical SAM domain protein** | **Open Ocean** | **Sargasso Sea, Bermuda** | **24** | **9.12e-18** |
| **radical SAM domain protein** | **Open Ocean** | **Sargasso Sea, Bermuda** | **24** | **9.43e-18** |
| **radical SAM** | **Hypersaline Lagoons** | **Galapagos Islands, Ecuador** | **31** | | **9.45e-18** |  | | --- | --- | |
| **radical SAM domain protein** | **Open Ocean** | **Sargasso Sea, Bermuda** | **28** | **2.14e-17** |
| **radical SAM domain protein** | **Open Ocean** | **Sargasso Sea, Bermuda** | **28** | **2.15e-17** |
| **radical SAM domain protein** | **Open Ocean** | **Sargasso Sea, Bermuda** | **28** | **2.17e-17** |
| Protein function | Metagenome | Location | % identity | e-value |
| **radical SAM domain protein** | **Open Ocean** | **Sargasso Sea, Bermuda** | **28** | **2.21e-17** |
| **radical SAM domain protein** | **Open Ocean** | **Sargasso Sea, Bermuda** | **28** | **2.25e-17** |
| **radical SAM domain protein** | **Open Ocean** | **Sargasso Sea, Bermuda** | **28** | **2.40e-17** |
| **radical SAM domain protein** | **Open Ocean** | **Sargasso Sea, Bermuda** | **24** | **2.46e-17** |
| **radical SAM domain protein** | **Open Ocean** | **Sargasso Sea, Bermuda** | **25** | **2.86e-17** |
| **radical SAM domain protein** | **Open Ocean** | **Sargasso Sea, Bermuda** | **29** | **2.98e-17** |
| **radical SAM domain protein** | **Open Ocean** | **Sargasso Sea, Bermuda** | **28** | **3.21e-17** |
| **radical SAM domain protein** | **Open Ocean** | **Sargasso Sea, Bermuda** | **28** | **4.09e-17** |
| **radical SAM domain protein** | **Open Ocean** | **Sargasso Sea, Bermuda** | **28** | **4.30e-17** |
| **radical SAM domain protein** | **Open Ocean** | **Sargasso Sea, Bermuda** | **28** | **7.53e-17** |
| **radical SAM domain protein** | **Open Ocean** | **Sargasso Sea, Bermuda** | **32** | **1.35e-16** |
| **radical SAM domain protein** | **Open Ocean** | **Sargasso Sea, Bermuda** | **28** | **1.57e-16** |
| **radical SAM domain protein** | **Open Ocean** | **Sargasso Sea, Bermuda** | **34** | **2.74e-16** |
| **radical SAM** | **Hypersaline Lagoons** | **Galapagos Islands, Ecuador** | **27** | **3.73e-16** |
| **radical SAM domain protein** | **Open Ocean** | **Sargasso Sea, Bermuda** | **32** | **3.96e-16** |
| **radical SAM domain protein** | **Mangrove** | **Galapagos Islands, Ecuador** | **32** | **4.30e-16** |
| **radical SAM** | **Hypersaline Lagoons** | **Galapagos Islands, Ecuador** | **31** | **1.52e-15** |
| **radical SAM domain protein** | **Open Ocean** | **Sargasso Sea, Bermuda** | **35** | **3.14e-14** |
| **radical SAM domain protein** | **Open Ocean** | **Sargasso Sea, Bermuda** | **33** | **4.07e-14** |
| **radical SAM domain protein** | **Open Ocean** | **Sargasso Sea, Bermuda** | **23** | **1.05e-13** |
| **radical SAM domain protein** | **Open Ocean** | **Sargasso Sea, Bermuda** | **31** | **1.07e-13** |
| **radical SAM domain protein** | **Open Ocean** | **Sargasso Sea, Bermuda** | **30** | **1.81e-13** |
| **radical SAM** | **Hypersaline Lagoons** | **Galapagos Islands, Ecuador** | **29** | **2.32e-13** |
| **radical SAM** | **Hypersaline Lagoons** | **Galapagos Islands, Ecuador** | **29** | **2.45e-13** |
| **radical SAM domain protein** | **Mangrove** | **Galapagos Islands, Ecuador** | **34** | **2.79e-13** |
| **radical SAM domain protein** | **Mangrove** | **Galapagos Islands, Ecuador** | **33** | **2.80e-13** |
| **radical SAM domain protein** | **Coastal Sample** | **Indian Ocean, Seychelles** | **30** | **3.27e-13** |
| **radical SAM domain protein** | **Open Ocean** | **Sargasso Sea, Bermuda** | **31** | **3.69e-13** |
| **radical SAM domain protein** | **Open Ocean** | **Sargasso Sea, Bermuda** | **31** | | **4.66e-13** |  | | --- | --- | |
| **radical SAM domain protein** | **Open Ocean** | **Sargasso Sea, Bermuda** | **33** | **5.24e-13** |
| **radical SAM domain protein** | **Open Ocean** | **Sargasso Sea, Bermuda** | **31** | **5.37e-13** |
| **radical SAM domain protein** | **Open Ocean** | **Sargasso Sea, Bermuda** | **31** | **5.37e-13** |
| **radical SAM domain protein** | **Open Ocean** | **Sargasso Sea, Bermuda** | **31** | **5.50e-13** |
| **radical SAM domain protein** | **Open Ocean** | **Sargasso Sea, Bermuda** | **31** | **5.50e-13** |
| **radical SAM domain protein** | **Open Ocean** | **Sargasso Sea, Bermuda** | **31** | **5.60e-13** |
| **radical SAM domain protein** | **Open Ocean** | **Sargasso Sea, Bermuda** | **31** | **5.84e-13** |
| **radical SAM domain protein** | **Open Ocean** | **Sargasso Sea, Bermuda** | **31** | **5.93e-13** |
| **radical SAM domain protein** | **Open Ocean** | **Sargasso Sea, Bermuda** | **31** | **6.08e-13** |
| **radical SAM domain protein** | **Open Ocean** | **Sargasso Sea, Bermuda** | **31** | **6.13e-13** |
| **radical SAM domain protein** | **Open Ocean** | **Sargasso Sea, Bermuda** | **31** | **6.29e-13** |
| **radical SAM domain protein** | **Open Ocean** | **Sargasso Sea, Bermuda** | **31** | **6.45e-13** |
| **radical SAM domain protein** | **Open Ocean** | **Sargasso Sea, Bermuda** | **31** | **7.13e-13** |
| **radical SAM domain protein** | **Open Ocean** | **Sargasso Sea, Bermuda** | **31** | **7.43e-13** |
| **radical SAM domain protein** | **Open Ocean** | **Sargasso Sea, Bermuda** | **31** | **7.86e-13** |
| **Heme Biosynthesis** | **Open Ocean** | **Caribbean Sea, Mexico** | **26** | **8.18e-13** |
| **radical SAM domain protein** | **Open Ocean** | **Sargasso Sea, Bermuda** | **31** | **8.28e-13** |
| **Heme Biosynthesis** | **Mangrove** | **Galapagos Islands, Ecuador** | **22** | **1.04e-12** |
| **radical SAM** | **Hypersaline Lagoons** | **Galapagos Islands, Ecuador** | **35** | **1.12e-12** |
| **radical SAM domain protein** | **Mangrove** | **Galapagos Islands, Ecuador** | **30** | **1.22e-12** |
| **radical SAM domain protein** | **Mangrove** | **Galapagos Islands, Ecuador** | **30** | **1.25e-12** |
| **radical SAM domain protein** | **Open Ocean** | **Indian Ocean, International** | **28** | **1.41e-12** |
| **radical SAM domain protein** | **Coastal upwelling** | **Galapagos Islands, Ecuador** | **35** | **1.44e-12** |
| **radical SAM domain protein** | **Surface soil** | **Minnesota, USA** | **29** | **1.46e-12** |
| **Putative arylsulfatase regulator** | **Silicate Sediments** | **Mediterranean Sea, Italy** | **32** | **1.48e-12** |
| **radical SAM** | **Hypersaline Lagoons** | **Galapagos Islands, Ecuador** | **26** | **1.70e-12** |
| **Heme Biosynthesis** | **Mangrove** | **Galapagos Islands, Ecuador** | **22** | **1.73e-12** |
| **Heme Biosynthesis** | **Open Ocean** | **Caribbean Sea, Mexico** | **27** | **1.78e-12** |
| **Hypothetical Protein** | **Coastal** | **North American East Coast, USA** | **34** | **2.03e-12** |
| **radical SAM domain protein** | **Mangrove** | **Galapagos Islands, Ecuador** | **31** | **2.68e-12** |
| **radical SAM domain protein** | **Acid Mine Drainage** | **Iron Mountain, California, USA** | **30** | **3.32e-12** |
| **radical SAM domain protein** | **Mangrove** | **Galapagos Islands, Ecuador** | **31** | **3.37e-12** |
| **radical SAM domain protein** | **Mangrove** | **Galapagos Islands, Ecuador** | **28** | **3.53e-12** |
| **radical SAM domain protein** | **Acid Mine Drainage** | **Iron Mountain, California, USA** | **30** | **3.55e-12** |
| **radical SAM domain protein** | **Acid Mine Drainage** | **Iron Mountain, California, USA** | **30** | **3.80e-12** |
| **radical SAM domain protein** | **Surface soil** | **Minnesota, USA** | **27** | **3.90e-12** |
| **radical SAM domain protein** | **Acid Mine Drainage** | **Iron Mountain, California, USA** | **30** | **4.31e-12** |
| **radical SAM domain protein** | **Surface soil** | **Minnesota, USA** | **30** | **4.31e-12** |
| **radical SAM domain protein** | **Open Ocean** | **Sargasso Sea, Bermuda** | **36** | **5.49e-12** |
| **radical SAM domain protein** | **Mangrove** | **Galapagos Islands, Ecuador** | **33** | **5.69e-12** |
| **radical SAM domain protein** | **Open Ocean** | **Sargasso Sea, Bermuda** | **32** | **7.92e-12** |
| **radical SAM domain protein** | **Acid Mine Drainage** | **Iron Mountain, California, USA** | **31** | **8.12e-12** |
| **Hypothetical Protein** | **Open Ocean** | **Caribbean sea, Mexico** | **27** | **9.51e-12** |
| **radical SAM domain protein** | **Open Ocean** | **Sargasso Sea, Bermuda** | **21** | **1.11e-11** |
| **radical SAM domain protein** | **Open Ocean** | **Sargasso Sea, Bermuda** | **37** | **1.27e-11** |
| Protein function | Metagenome | Location | % identity | e-value |
| **radical SAM domain protein** | **Open Ocean** | **Sargasso Sea, Bermuda** | **31** | **1.29e-11** |
| **radical SAM domain protein** | **Open Ocean** | **Sargasso Sea, Bermuda** | **31** | **1.31e-11** |
| **radical SAM domain protein** | **Open Ocean** | **Sargasso Sea, Bermuda** | **31** | **1.36e-11** |
| **radical SAM domain protein** | **Open Ocean** | **Sargasso Sea, Bermuda** | **31** | **1.46e-11** |
| **radical SAM domain protein** | **Open Ocean** | **Sargasso Sea, Bermuda** | **31** | **1.66e-11** |
| **radical SAM domain protein** | **Open Ocean** | **Sargasso Sea, Bermuda** | **31** | **1.69e-11** |
| **radical SAM domain protein** | **Open Ocean** | **Sargasso Sea, Bermuda** | **31** | **1.74e-11** |
| **radical SAM domain protein** | **Open Ocean** | **Sargasso Sea, Bermuda** | **31** | **1.78e-11** |
| **Hypothetical Protein** | **Coastal** | **North American East Coast, USA** | **37** | **1.79e-11** |
| **radical SAM domain protein** | **Open Ocean** | **Sargasso Sea, Bermuda** | **29** | **1.87e-11** |
| **radical SAM domain protein** | **Open Ocean** | **Sargasso Sea, Bermuda** | **31** | **2.03e-11** |
| **radical SAM domain protein** | **Open Ocean** | **Sargasso Sea, Bermuda** | **31** | **2.09e-11** |
| **radical SAM domain protein** | **Mangrove** | **Galapagos Islands, Ecuador** | **35** | **2.09e-11** |
| **radical SAM domain protein** | **Open Ocean** | **Sargasso Sea, Bermuda** | **31** | **2.21e-11** |
| **radical SAM domain protein** | **Open Ocean** | **Sargasso Sea, Bermuda** | **31** | **2.25e-11** |
| **radical SAM domain protein** | **Open Ocean** | **Sargasso Sea, Bermuda** | **31** | **2.29e-11** |
| **radical SAM domain protein** | **Open Ocean** | **Sargasso Sea, Bermuda** | **31** | **2.31e-11** |
| **radical SAM domain protein** | **Open Ocean** | **Sargasso Sea, Bermuda** | **29** | **2.38e-11** |
| **radical SAM domain protein** | **Acid Mine Drainage** | **Iron Mountain, California, USA** | **26** | **2.40e-11** |
| **radical SAM domain protein** | **Open Ocean** | **Sargasso Sea, Bermuda** | **31** | **2.40e-11** |
| **radical SAM domain protein** | **Open Ocean** | **Sargasso Sea, Bermuda** | **31** | **2.44e-11** |
| **radical SAM domain protein** | **Open Ocean** | **Sargasso Sea, Bermuda** | **28** | **2.51e-11** |
| **radical SAM domain protein** | **Open Ocean** | **Sargasso Sea, Bermuda** | **31** | **2.59e-11** |
| **radical SAM domain protein** | **Acid Mine Drainage** | **Iron Mountain, California, USA** | **26** | **2.91e-11** |
| **radical SAM domain protein** | **Acid Mine Drainage** | **Iron Mountain, California, USA** | **31** | **2.91e-11** |
| **Hypothetical Protein** | **Coastal Upwelling** | **Galapagos Islands, Ecuador** | **35** | **2.96e-11** |
| **radical SAM domain protein** | **Acid Mine Drainage** | **Iron Mountain, California, USA** | **26** | **3.01e-11** |
| **radical SAM domain protein** | **Open Ocean** | **Sargasso Sea, Bermuda** | **29** | **3.33e-11** |
| **radical SAM domain protein** | **Acid Mine Drainage** | **Iron Mountain, California, USA** | **26** | **3.35e-11** |
| **radical SAM domain protein** | **Open Ocean** | **Sargasso Sea, Bermuda** | **29** | **3.44e-11** |
| **radical SAM domain protein** | **Open Ocean** | **Sargasso Sea, Bermuda** | **29** | **3.47e-11** |
| **radical SAM domain protein** | **Open Ocean** | **Sargasso Sea, Bermuda** | **29** | **3.62e-11** |
| **radical SAM** | **Hypersaline Lagoons** | **Galapagos Islands, Ecuador** | **24** | **3.62e-11** |
| **radical SAM domain protein** | **Acid Mine Drainage** | **Iron Mountain, California, USA** | **32** | **3.77e-11** |
| **radical SAM** | **Hypersaline Lagoons** | **Galapagos Islands, Ecuador** | **24** | **3.78e-11** |
| **radical SAM domain protein** | **Acid Mine Drainage** | **Iron Mountain, California, USA** | **31** | **3.80e-11** |
| **radical SAM domain protein** | **Open Ocean** | **Sargasso Sea, Bermuda** | **29** | **3.87e-11** |
| **radical SAM domain protein** | **Open Ocean** | **Sargasso Sea, Bermuda** | **29** | **3.93e-11** |
| **radical SAM domain protein** | **Open Ocean** | **Sargasso Sea, Bermuda** | **29** | **3.93e-11** |
| **radical SAM domain protein** | **Acid Mine Drainage** | **Iron Mountain, California, USA** | **26** | **4.02e-11** |
| **radical SAM domain protein** | **Open Ocean** | **Sargasso Sea, Bermuda** | **29** | **4.07e-11** |
| **radical SAM domain protein** | **Open Ocean** | **Sargasso Sea, Bermuda** | **29** | **4.17e-11** |
| **radical SAM domain protein** | **Acid Mine Drainage** | **Iron Mountain, California, USA** | **27** | **4.23e-11** |
| **radical SAM domain protein** | **Acid Mine Drainage** | **Iron Mountain, California, USA** | **27** | **4.49e-11** |
| **radical SAM domain protein** | **Acid Mine Drainage** | **Iron Mountain, California, USA** | **27** | **5.08e-11** |
| **radical SAM domain protein** | **Acid Mine Drainage** | **Iron Mountain, California, USA** | **27** | **5.17e-11** |
| **radical SAM domain protein** | **Mangrove** | **Galapagos Islands, Ecuador** | **24** | **5.22e-11** |
| **radical SAM domain protein** | **Mangrove** | **Galapagos Islands, Ecuador** | **32** | **5.65e-11** |
| **radical SAM domain protein** | **Acid Mine Drainage** | **Iron Mountain, California, USA** | **27** | **6.32e-11** |
| **radical SAM** | **Hypersaline Lagoons** | **Galapagos Islands, Ecuador** | **33** | **6.62e-11** |
| **radical SAM domain protein** | **Mangrove** | **Galapagos Islands, Ecuador** | **25** | **7.99e-11** |
| **radical SAM domain protein** | **Open Ocean** | **Indian Ocean, International** | **26** | **8.54e-11** |
| **radical SAM domain protein** | **Surface Soil** | **Minnesota, USA** | **30** | **8.79e-11** |
| **radical SAM domain protein** | **Surface Soil** | **Minnesota, USA** | **29** | **9.21e-11** |
| **radical SAM domain protein** | **Open Ocean** | **Sargasso Sea, Bermuda** | **31** | **9.76e-11** |
| **radical SAM** | **Hypersaline Lagoons** | **Galapagos Islands, Ecuador** | **31** | **1.10e-10** |
| **radical SAM** | **Hypersaline Lagoons** | **Galapagos Islands, Ecuador** | **31** | **1.11e-10** |
| **radical SAM domain protein** | **Open Ocean** | **Sargasso Sea, Bermuda** | **31** | **1.31e-10** |
| **radical SAM domain protein** | **Acid Mine Drainage** | **Iron Mountain, California, USA** | **25** | **1.32e-10** |
| **radical SAM domain protein** | **Acid Mine Drainage** | **Iron Mountain, California, USA** | **25** | **1.39e-10** |
| **Arylsulfatase regulator** | **Silicate Sediments** | **Mediterranean Sea, Italy** | **31** | **1.49e-10** |
| **radical SAM domain protein** | **Acid Mine Drainage** | **Iron Mountain, California, USA** | **29** | **1.52e-10** |
| **radical SAM domain protein** | **Open Ocean** | **Sargasso Sea, Bermuda** | **26** | **1.53e-10** |
| **radical SAM domain protein** | **Open Ocean** | **Sargasso Sea, Bermuda** | **29** | **1.57e-10** |
| **radical SAM domain protein** | **Open Ocean** | **Sargasso Sea, Bermuda** | **27** | **1.58e-10** |
| **radical SAM domain protein** | **Open Ocean** | **Sargasso Sea, Bermuda** | **31** | **1.64e-10** |
| **radical SAM domain protein** | **Open Ocean** | **Sargasso Sea, Bermuda** | **31** | **1.68e-10** |
| **radical SAM** | **Hypersaline Lagoons** | **Galapagos Islands, Ecuador** | **29** | **1.69e-10** |
| **radical SAM domain protein** | **Open Ocean** | **Sargasso Sea, Bermuda** | **26** | **1.69e-10** |
| **radical SAM domain protein** | **Open Ocean** | **Sargasso Sea, Bermuda** | **31** | **1.72e-10** |
| **radical SAM domain protein** | **Open Ocean** | **Sargasso Sea, Bermuda** | **29** | **1.72e-10** |
| **radical SAM domain protein** | **Open Ocean** | **Sargasso Sea, Bermuda** | **27** | **1.72e-10** |
| **radical SAM domain protein** | **Open Ocean** | **Sargasso Sea, Bermuda** | **31** | **1.75e-10** |
| **radical SAM domain protein** | **Open Ocean** | **Sargasso Sea, Bermuda** | **27** | **1.78e-10** |
| **radical SAM domain protein** | **Open Ocean** | **Sargasso Sea, Bermuda** | **31** | **1.82e-10** |
| **radical SAM domain protein** | **Acid Mine Drainage** | **Iron Mountain, California, USA** | **29** | **2.60e-10** |
| Protein function | Metagenome | Location | % identity | e-value |
| **radical SAM domain protein** | **Open Ocean** | **Sargasso Sea, Bermuda** | **27** | **2.61e-10** |
| **radical SAM domain protein** | **Open Ocean** | **Sargasso Sea, Bermuda** | **25** | **2.96e-10** |
| **radical SAM domain protein** | **Mangrove** | **Galapagos Islands, Ecuador** | **28** | **3.04e-10** |
| **radical SAM domain protein** | **Open Ocean** | **Sargasso Sea, Bermuda** | **26** | **3.11e-10** |
| **radical SAM domain protein** | **Acid Mine Drainage** | **Iron Mountain, California, USA** | **25** | **3.31e-10** |
| **radical SAM domain protein** | **Acid Mine Drainage** | **Iron Mountain, California, USA** | **25** | **3.36e-10** |
| **radical SAM domain protein** | **Acid Mine Drainage** | **Iron Mountain, California, USA** | **25** | **3.40e-10** |
| **Putative arylsulfatase regulatory protein** | **Open Ocean** | **Sargasso Sea, Bermuda** | **27** | **3.41e-10** |
| **radical SAM domain protein** | **Acid Mine Drainage** | **Iron Mountain, California, USA** | **25** | **3.44e-10** |
| **radical SAM domain protein** | **Acid Mine Drainage** | **Iron Mountain, California, USA** | **25** | **3.45e-10** |
| **radical SAM domain protein** | **Acid Mine Drainage** | **Iron Mountain, California, USA** | **25** | **3.53e-10** |
| **Putative arylsulfatase regulatory protein** | **Open Ocean** | **Sargasso Sea, Bermuda** | **27** | **3.55e-10** |
| **radical SAM domain protein** | **Acid Mine Drainage** | **Iron Mountain, California, USA** | **25** | **3.78e-10** |
| **radical SAM domain protein** | **Open Ocean** | **Sargasso Sea, Bermuda** | **26** | **3.80e-10** |
| **radical SAM domain protein** | **Open Ocean** | **Sargasso Sea, Bermuda** | **26** | **3.83e-10** |
| **radical SAM domain protein** | **Open Ocean** | **Sargasso Sea, Bermuda** | **26** | **4.13e-10** |
| **radical SAM domain protein** | **Acid Mine Drainage** | **Iron Mountain, California, USA** | **32** | **4.15e-10** |
| **radical SAM domain protein** | **Mangrove** | **Galapagos Islands, Ecuador** | **25** | **4.16e-10** |
| **radical SAM domain protein** | **Acid Mine Drainage** | **Iron Mountain, California, USA** | **25** | **4.21e-10** |
| **radical SAM domain protein** | **Open Ocean** | **Sargasso Sea, Bermuda** | **26** | **4.49e-10** |
| **radical SAM domain protein** | **Open Ocean** | **Sargasso Sea, Bermuda** | **26** | **4.60e-10** |
| **radical SAM domain protein** | **Open Ocean** | **Sargasso Sea, Bermuda** | **26** | **4.64e-10** |
| **radical SAM domain protein** | **Open Ocean** | **Sargasso Sea, Bermuda** | **26** | **4.72e-10** |
| **radical SAM** | **Hypersaline Lagoons** | **Galapagos Islands, Ecuador** | **28** | **4.75e-10** |
| **radical SAM domain protein** | **Open Ocean** | **Sargasso Sea, Bermuda** | **26** | **5.09e-10** |
| **radical SAM domain protein** | **Open Ocean** | **Sargasso Sea, Bermuda** | **28** | **5.35e-10** |
| **radical SAM** | **Acid Mine Drainage** | **Iron Mountain, California, USA** | **32** | **5.79e-10** |
| **radical SAM domain protein** | **Open Ocean** | **Sargasso Sea, Bermuda** | **26** | **6.87e-10** |
| **radical SAM** | **Acid Mine Drainage** | **Iron Mountain, California, USA** | **29** | **7.08e-10** |
| **radical SAM domain protein** | **Open Ocean** | **Sargasso Sea, Bermuda** | **27** | **7.91e-10** |
| **radical SAM domain protein** | **Open Ocean** | **Sargasso Sea, Bermuda** | **27** | **8.46e-10** |
| **radical SAM domain protein** | **Mangrove** | **Galapagos Islands, Ecuador** | **31** | **9.53e-10** |
| **radical SAM domain protein** | **Mangrove** | **Galapagos Islands, Ecuador** | **26** | **1.01e-9** |
| **radical SAM domain protein** | **Mangrove** | **Galapagos Islands, Ecuador** | **30** | **1.08e-9** |
| **radical SAM domain protein** | **Mangrove** | **Galapagos Islands, Ecuador** | **30** | **1.10 e-9** |
| **radical SAM domain protein** | **Open Ocean** | **Sargasso Sea, Bermuda** | **30** | **1.16 e-9** |
| **radical SAM domain protein** | **Open Ocean** | **Sargasso Sea, Bermuda** | **27** | **1.17 e-9** |
| **radical SAM domain protein** | **Mangrove** | **Galapagos Islands, Ecuador** | **30** | **1.18 e-9** |
| **Putative arylsulfatase regulatory protein** | **Harbor** | **Indian Ocean, Tanzania** | **28** | **1.20 e-9** |
| **radical SAM domain protein** | **Open Ocean** | **Sargasso Sea, Bermuda** | **25** | **1.21 e-9** |
| **radical SAM domain protein** | **Open Ocean** | **Sargasso Sea, Bermuda** | **26** | **1.24 e-9** |
| **Hypothetical protein** | **Coral reef Atoll** | **Polynesia Archipelagos, French Polynesia** | **37** | **1.36 e-9** |
| **radical SAM domain protein** | **Mangrove** | **Galapagos Islands, Ecuador** | **28** | **1.49 e-9** |
| **radical SAM domain protein** | **Open Ocean** | **Sargasso Sea, Bermuda** | **26** | **1.49 e-9** |
| **radical SAM domain protein** | **Open Ocean** | **Sargasso Sea, Bermuda** | **25** | **1.52 e-9** |
| **radical SAM domain protein** | **Open Ocean** | **Sargasso Sea, Bermuda** | **25** | **1.56 e-9** |
| **radical SAM domain protein** | **Open Ocean** | **Sargasso Sea, Bermuda** | **28** | **1.69 e-9** |
| **radical SAM domain protein** | **Open Ocean** | **Sargasso Sea, Bermuda** | **26** | **1.73 e-9** |
| **radical SAM domain protein** | **Open Ocean** | **Sargasso Sea, Bermuda** | **26** | **1.88 e-9** |
| **radical SAM domain protein** | **Open Ocean** | **Sargasso Sea, Bermuda** | **26** | **2.01 e-9** |
| **radical SAM** | **Hypersaline Lagoons** | **Galapagos Islands, Ecuador** | **28** | **2.04 e-9** |
| **radical SAM domain protein** | **Open Ocean** | **Sargasso Sea, Bermuda** | **26** | **2.12 e-9** |
| **Arylsulfatase regulator** | **Surface soil** | **Minnesota, USA** | **33** | **2.13 e-9** |
| **radical SAM domain protein** | **Open Ocean** | **Sargasso Sea, Bermuda** | **26** | **2.19 e-9** |
| **radical SAM domain protein** | **Open Ocean** | **Sargasso Sea, Bermuda** | **27** | **2.26 e-9** |
| **Arylsulfatase regulator** | **Surface soil** | **Minnesota, USA** | **33** | **2.74 e-9** |
| **radical SAM domain protein** | **Open Ocean** | **Sargasso Sea, Bermuda** | **26** | **3.14 e-9** |
| **radical SAM** | **Hypersaline Lagoons** | **Galapagos Islands, Ecuador** | **31** | **4.29 e-9** |
| **Heme biosynthesis** | **Open Ocean** | **Caribbean Sea, Mexico** | **26** | **4.51 e-9** |
| **radical SAM domain protein** | **Open Ocean** | **Sargasso Sea, Bermuda** | **22** | **4.66 e-9** |
| **radical SAM domain protein** | **Open Ocean** | **Sargasso Sea, Bermuda** | **22** | **5.31 e-9** |
| **radical SAM domain protein** | **Open Ocean** | **Sargasso Sea, Bermuda** | **28** | **2.00 e-8** |
| **radical SAM** | **Hypersaline Lagoons** | **Galapagos Islands, Ecuador** | **29** | **2.28 e-8** |
| **radical SAM** | **Hypersaline Lagoons** | **Galapagos Islands, Ecuador** | **29** | **2.33 e-8** |
| **Arylsulfatase regulator** | **Surface soil** | **Minnesota, USA** | **30** | **2.37 e-8** |
| **radical SAM** | **Hypersaline Lagoons** | **Galapagos Islands, Ecuador** | **27** | **2.57 e-8** |
| **radical SAM domain protein** | **Open Ocean** | **Sargasso Sea, Bermuda** | **22** | **2.73 e-8** |
| **radical SAM domain protein** | **Open Ocean** | **Sargasso Sea, Bermuda** | **22** | **2.88 e-8** |
| **radical SAM domain protein** | **Open Ocean** | **Sargasso Sea, Bermuda** | **30** | **3.31 e-8** |
| **radical SAM domain protein** | **Open Ocean** | **Sargasso Sea, Bermuda** | **30** | **3.42 e-8** |
| **radical SAM domain protein** | **Open Ocean** | **Sargasso Sea, Bermuda** | **21** | **3.45 e-8** |
| **radical SAM** | **Hypersaline Lagoons** | **Galapagos Islands, Ecuador** | **26** | **3.54 e-8** |
| **radical SAM domain protein** | **Open Ocean** | **Sargasso Sea, Bermuda** | **21** | **3.56 e-8** |
| **radical SAM domain protein** | **Open Ocean** | **Sargasso Sea, Bermuda** | **21** | **3.56 e-8** |
| **radical SAM domain protein** | **Open Ocean** | **Sargasso Sea, Bermuda** | **21** | **3.76 e-8** |
| **radical SAM domain protein** | **Open Ocean** | **Galapagos Islands, Ecuador** | **28** | **4.08 e-8** |
| Protein function | Metagenome | Location | % identity | e-value |
| **radical SAM domain protein** | **Open Ocean** | **Sargasso Sea, Bermuda** | **22** | **4.14 e-8** |
| **radical SAM domain protein** | **Open Ocean** | **Sargasso Sea, Bermuda** | **30** | **4.14 e-8** |
| **radical SAM domain protein** | **Open Ocean** | **Sargasso Sea, Bermuda** | **30** | **4.29 e-8** |
| **radical SAM** | **Acid Mine Drainage** | **Iron Mountain, California, USA** | **30** | **4.29 e-8** |
| **radical SAM domain protein** | **Open Ocean** | **Sargasso Sea, Bermuda** | **22** | **4.40 e-8** |
| **radical SAM domain protein** | **Open Ocean** | **Galapagos Islands, Ecuador** | **28** | **4.85 e-8** |
| **radical SAM** | **Acid Mine Drainage** | **Iron Mountain, California, USA** | **30** | **4.86 e-8** |
| **radical SAM** | **Acid Mine Drainage** | **Iron Mountain, California, USA** | **30** | **5.11 e-8** |
| **radical SAM** | **Acid Mine Drainage** | **Iron Mountain, California, USA** | **30** | **5.24 e-8** |
| **radical SAM domain protein** | **Open Ocean** | **Sargasso Sea, Bermuda** | **28** | **5.49 e-8** |
| **radical SAM domain protein** | **Mangrove** | **Galapagos Islands, Ecuador** | **28** | **5.67 e-8** |
| **radical SAM domain protein** | **Open Ocean** | **Sargasso Sea, Bermuda** | **27** | **5.79 e-8** |
| **radical SAM domain protein** | **Open Ocean** | **Sargasso Sea, Bermuda** | **27** | **6.33 e-8** |
| **radical SAM** | **Acid Mine Drainage** | **Iron Mountain, California, USA** | **30** | **6.35 e-8** |
| **radical SAM domain protein** | **Mangrove** | **Galapagos Islands, Ecuador** | **25** | **6.51 e-8** |
| **radical SAM domain protein** | **Coastal** | **North American East Coast, USA** | **21** | **6.73 e-8** |
| **radical SAM domain protein** | **Coastal upwelling** | **Galapagos Islands, Ecuador** | **30** | **6.90 e-8** |
| **radical SAM domain protein** | **Surface soil** | **Minnesota, USA** | **29** | **7.02 e-8** |
| **radical SAM domain protein** | **Coastal upwelling** | **Galapagos Islands, Ecuador** | **29** | **7.26 e-8** |
| **radical SAM domain protein** | **Fresh water** | **Panama canal, Panama** | **25** | **1.39 e-7** |
| **Heme biosynthesis** | **Open Ocean** | **Caribbean Sea, Mexico** | **25** | **1.48 e-7** |
| **radical SAM** | **Acid Mine Drainage** | **Iron Mountain, California, USA** | **30** | **1.55 e-7** |
| **radical SAM domain protein** | **Open Ocean** | **Sargasso Sea, Bermuda** | **27** | **1.73 e-7** |
| **radical SAM domain protein** | **Open Ocean** | **Sargasso Sea, Bermuda** | **33** | **2.05 e-7** |
| **radical SAM domain protein** | **Mangrove** | **Galapagos Islands, Ecuador** | **22** | **2.15 e-7** |
| **radical SAM domain protein** | **Open Ocean** | **Sargasso Sea, Bermuda** | **25** | **2.35 e-7** |
| **radical SAM domain protein** | **Surface soil** | **Minnesota, USA** | **25** | **2.59 e-7** |
| **radical SAM domain protein** | **Coastal Sample** | **Indian Ocean, Seychelles** | **32** | **2.78 e-7** |
| **radical SAM** | **Acid Mine Drainage** | **Iron Mountain, California, USA** | **28** | **2.85 e-7** |
| **radical SAM** | **Acid Mine Drainage** | **Iron Mountain, California, USA** | **30** | **2.92 e-7** |
| **radical SAM domain protein** | **Coastal upwelling** | **Galapagos Islands, Ecuador** | **28** | **2.94 e-7** |
| **radical SAM domain protein** | **Coastal** | **Caribbean Sea, USA** | **30** | **2.97 e-7** |
| **radical SAM** | **Acid Mine Drainage** | **Iron Mountain, California, USA** | **41** | **3.84 e-7** |
| **radical SAM domain protein** | **Coastal upwelling** | **Galapagos Islands, Ecuador** | **32** | **4.29 e-7** |
| **radical SAM domain protein** | **Coastal upwelling** | **Galapagos Islands, Ecuador** | **31** | **4.30 e-7** |
| **radical SAM** | **Acid Mine Drainage** | **Iron Mountain, California, USA** | **41** | **5.10 e-7** |
| **radical SAM domain protein** | **Open Ocean** | **Sargasso Sea, Bermuda** | **31** | **5.89 e-7** |
| **radical SAM** | **Acid Mine Drainage** | **Iron Mountain, California, USA** | **41** | **6.17 e-7** |
| **radical SAM domain protein** | **Open Ocean** | **Sargasso Sea, Bermuda** | **25** | **6.75 e-7** |
| **radical SAM domain protein** | **Surface soil** | **Minnesota, USA** | **28** | **6.86 e-7** |
| **radical SAM domain protein** | **Open Ocean** | **Sargasso Sea, Bermuda** | **25** | **7.28 e-7** |
| **radical SAM** | **Acid Mine Drainage** | **Iron Mountain, California, USA** | **41** | **7.86 e-7** |
| **radical SAM** | **Acid Mine Drainage** | **Iron Mountain, California, USA** | **28** | **7.93 e-7** |
| **radical SAM** | **Acid Mine Drainage** | **Iron Mountain, California, USA** | **41** | **8.27 e-7** |
| **radical SAM domain protein** | **Coastal** | **Caribbean Sea, USA** | **32** | **8.45 e-7** |
| **radical SAM domain protein** | **Mangrove** | **Galapagos Islands, Ecuador** | **30** | **8.58 e-7** |
| **radical SAM domain protein** | **Open Ocean** | **Sargasso Sea, Bermuda** | **25** | **8.82 e-7** |
| **radical SAM domain protein** | **Open Ocean** | **Sargasso Sea, Bermuda** | **22** | **9.25 e-7** |
| **radical SAM** | **Acid Mine Drainage** | **Iron Mountain, California, USA** | **28** | **9.29 e-7** |
| **radical SAM** | **Acid Mine Drainage** | **Iron Mountain, California, USA** | **28** | **9.29 e-7** |
| **radical SAM** | **Acid Mine Drainage** | **Iron Mountain, California, USA** | **28** | **1.027 e-6** |
| **radical SAM domain protein** | **Open Ocean** | **Sargasso Sea, Bermuda** | **26** | **1.041 e-6** |
| **putative arylsulfatase regulator** | **Mediterranean Sea** | **Italy** | **23** | **1.045 e-6** |
| **radical SAM domain protein** | **Open Ocean** | **Sargasso Sea, Bermuda** | **25** | **1.095 e-6** |
| **arylsulfatase-activating protein AtsB** | **Hypersaline** | **Galapagos Islands, Ecuador** | **25** | **1.412 e-6** |
| **radical SAM** | **Acid Mine Drainage** | **Iron Mountain, California, USA** | **28** | **1.422 e-6** |
| **Heme biosynthesis** | **Open Ocean** | **Caribbean Sea, Mexico** | **25** | **1.747 e-6** |
| **radical SAM** | **Acid Mine Drainage** | **Iron Mountain, California, USA** | **41** | **1.752 e-6** |
| **radical SAM domain protein** | **Open Ocean** | **Sargasso Sea, Bermuda** | **26** | **2.081 e-6** |
| **radical SAM domain protein** | **Open Ocean** | **Sargasso Sea, Bermuda** | **26** | **2.081 e-6** |
| **radical SAM domain protein** | **Open Ocean** | **Sargasso Sea, Bermuda** | **23** | **2.166 e-6** |
| **radical SAM domain protein** | **Open Ocean** | **Sargasso Sea, Bermuda** | **26** | **2.188 e-6** |
| **radical SAM domain protein** | **Open Ocean** | **Sargasso Sea, Bermuda** | **35** | **2.25 e-6** |
| **radical SAM** | **Acid Mine Drainage** | **Iron Mountain, California, USA** | **36** | **2.487 e-6** |
| **radical SAM domain protein** | **Open Ocean** | **Sargasso Sea, Bermuda** | **26** | **2.586 e-6** |
| **radical SAM domain protein** | **Open Ocean** | **Sargasso Sea, Bermuda** | **26** | **2.607 e-6** |
| **radical SAM domain protein** | **Open Ocean** | **Sargasso Sea, Bermuda** | **26** | **2.696 e-6** |
| **radical SAM domain protein** | **Coastal sample** | **Indian Ocean, Seychelles** | **27** | **2.796 e-6** |
| **radical SAM domain protein** | **Open Ocean** | **Sargasso Sea, Bermuda** | **26** | **2.81 e-6** |
| **radical SAM domain protein** | **Open Ocean** | **Sargasso Sea, Bermuda** | **26** | **31.59 e-6** |
| **radical SAM** | **Acid Mine Drainage** | **Iron Mountain, California, USA** | **29** | **3.206 e-6** |
| **radical SAM domain protein** | **Open Ocean** | **Sargasso Sea, Bermuda** | **26** | **3.266 e-6** |
| **radical SAM domain protein** | **Open Ocean** | **Sargasso Sea, Bermuda** | **26** | **3.55 e-6** |
| **radical SAM domain protein** | **Open Ocean** | **Sargasso Sea, Bermuda** | **26** | **3.58 e-6** |
| Protein function | Metagenome | Location | % identity | e-value |
| **radical SAM domain protein** | **Open Ocean** | **Sargasso Sea, Bermuda** | **26** | **3.61 e-6** |
| **radical SAM** | **Acid Mine Drainage** | **Iron Mountain, California, USA** | **40** | **3.903 e-6** |
| **radical SAM domain protein** | **Open Ocean** | **Sargasso Sea, Bermuda** | **26** | **4.612 e-6** |
| **radical SAM domain protein** | **Open Ocean** | **Sargasso Sea, Bermuda** | **40** | **4.729 e-6** |
| **Arylsulfatase regulator** | **Hypersaline** | **Galapagos Islands, Ecuador** | **28** | **5.275 e-6** |
| **radical SAM domain protein** | **Open Ocean** | **Sargasso Sea, Bermuda** | **26** | **5.404 e-6** |
| **radical SAM domain protein** | **Open Ocean** | **Sargasso Sea, Bermuda** | **26** | **5.495 e-6** |
| **radical SAM** | **Acid Mine Drainage** | **Iron Mountain, California, USA** | **33** | **5.912 e-6** |
| **radical SAM domain protein** | **Open Ocean** | **Sargasso Sea, Bermuda** | **25** | **6.198 e-6** |
| **radical SAM domain protein** | **Surface Soil** | **Minnesota, USA** | **24** | **6.198 e-6** |
| **radical SAM domain protein** | **Open Ocean** | **Sargasso Sea, Bermuda** | **28** | **6.198 e-6** |
| **Hypothetical protein** | **Coastal** | **North American East Coast, USA** | **27** | **6.409 e-6** |
| **radical SAM domain protein** | **Fresh water** | **Panama Canal, Panama** | **26** | **6.553 e-6** |
| **radical SAM** | **Acid Mine Drainage** | **Iron Mountain, California, USA** | **33** | **8.393 e-6** |
| **Arylsulfatase regulator** | **Hypersaline** | **Galapagos Islands, Ecuador** | **34** | **9.2 e-6** |
| **radical SAM** | **Acid Mine Drainage** | **Iron Mountain, California, USA** | **33** | **9.672 e-6** |
