# Supplementary material for: Genome Mining for Radical SAM Protein Determinants Reveals Multiple Sactibiotic-Like Gene Clusters
Source: PLoS One. 2011 Jul 8;6(7):e20852. doi: 10.1371/journal.pone.0020852 (PMC3132745; doi:10.1371/journal.pone.0020852)
Supplement: Table S4 — TrnD homologs in metagenomic databases. (DOC) [file pone.0020852.s004.doc]

**Suppl Table 4** Trn D homologs in metagenomic databases

| Protein function | Metagenome | Location | % identity | e-value |
| --- | --- | --- | --- | --- |
| **radical SAM** | **Hypersaline Lagoons** | **Galapagos Islands, Ecuador** | **36** | **2.91 e-23** |
| **radical SAM domain protein** | **Mangrove** | **Galapagos Islands, Ecuador** | **38** | **8.54 e-21** |
| **radical SAM domain protein** | **Mangrove** | **Galapagos Islands, Ecuador** | **33** | **6.04 e-19** |
| **radical SAM domain protein** | **Mangrove** | **Galapagos Islands, Ecuador** | **33** | **6.20 e-19** |
| **radical SAM domain protein** | **Mangrove** | **Galapagos Islands, Ecuador** | **34** | **1.19 e-18** |
| **radical SAM** | **Hypersaline Lagoons** | **Galapagos Islands, Ecuador** | **31** | **1.83 e-18** |
| **radical SAM** | **Hypersaline Lagoons** | **Galapagos Islands, Ecuador** | **35** | **2.01 e-18** |
| **radical SAM domain protein** | **Open Ocean** | **Sargasso Sea, Bermuda** | **33** | **4.33 e-18** |
| **radical SAM domain protein** | **Open Ocean** | **Sargasso Sea, Bermuda** | **33** | **4.71 e-18** |
| **radical SAM** | **Acid Mine Drainage** | **Iron Mountain, California, USA** | **25** | **3.62 e-17** |
| **radical SAM** | **Acid Mine Drainage** | **Iron Mountain, California, USA** | **35** | **7.94 e-17** |
| **radical SAM** | **Acid Mine Drainage** | **Iron Mountain, California, USA** | **35** | **1.38 e-16** |
| **radical SAM** | **Acid Mine Drainage** | **Iron Mountain, California, USA** | **35** | **1.45 e-16** |
| **radical SAM domain protein** | **Open Ocean** | **Sargasso Sea, Bermuda** | **34** | **2.07 e-16** |
| **radical SAM domain protein** | **Silicate Sediments** | **Mediterranean Sea, Italy** | **30** | **3.36 e-16** |
| **radical SAM** | **Acid Mine Drainage** | **Iron Mountain, California, USA** | **29** | **1.22 e-15** |
| **Hypothetical protein** | **Coral reef** | **Polynesia Archipelagos, French Polynesia** | **26** | **6.45 e-15** |
| **radical SAM domain protein** | **Hydrothermal vent** | **Eastern Pacific Ocean, International** | **30** | **1.14 e-14** |
| **radical SAM domain protein** | **Surface soil** | **Minnesota, USA** | **29** | **2.09 e-14** |
| **radical SAM domain protein** | **Open Ocean** | **Sargasso Sea, Bermuda** | **26** | **8.29 e-14** |
| **radical SAM** | **Hypersaline Lagoons** | **Galapagos Islands, Ecuador** | **29** | **1.96 e-13** |
| **radical SAM domain protein** | **Open Ocean** | **Sargasso Sea, Bermuda** | **31** | **5.90 e-13** |
| **radical SAM** | **Acid Mine Drainage** | **Iron Mountain, California, USA** | **24** | **6.04 e-13** |
| **Heme biosynthesis** | **Open Ocean** | **Caribbean Sea, Mexico** | **25** | **6.72 e-13** |
| **Putative arylsulfatase regulator** | **Silicate Sediments** | **Mediterranean Sea, Italy** | **31** | **7.81 e-13** |
| **radical SAM domain protein** | **Mangrove** | **Galapagos Islands, Ecuador** | **30** | **1.11 e-12** |
| **Putative arylsulfatase regulator** | **Silicate Sediments** | **Mediterranean Sea, Italy** | **25** | **1.70 e-12** |
| **radical SAM** | **Acid Mine Drainage** | **Iron Mountain, California, USA** | **30** | **2.20 e-12** |
| **radical SAM** | **Acid Mine Drainage** | **Iron Mountain, California, USA** | **31** | **2.29 e-12** |
| **radical SAM** | **Acid Mine Drainage** | **Iron Mountain, California, USA** | **31** | **2.35 e-12** |
| **radical SAM** | **Acid Mine Drainage** | **Iron Mountain, California, USA** | **30** | **2.49e-12** |
| **radical SAM** | **Acid Mine Drainage** | **Iron Mountain, California, USA** | **37** | **2.85e-12** |
| **Arylsulfatase regulator** | **Sewage Sludge** | **Brisbane, Queensland, Australia** | **29** | **3.34e-12** |
| **radical SAM** | **Acid Mine Drainage** | **Iron Mountain, California, USA** | **31** | **3.51e-12** |
| **radical SAM domain protein** | **Surface soil** | **Minnesota, USA** | **27** | **3.55e-12** |
| **Radical SAM** | **Sewage Sludge** | **Madison, Wisconsin, USA** | **23** | **7.00e-12** |
| **radical SAM** | **Acid Mine Drainage** | **Iron Mountain, California, USA** | **25** | **1.31e-11** |
| **radical SAM** | **Acid Mine Drainage** | **Iron Mountain, California, USA** | **32** | **1.63e-11** |
| **Radical SAM** | **Sewage Sludge** | **Madison, Wisconsin, USA** | **24** | **1.65e-11** |
| **Radical SAM** | **Sewage Sludge** | **Madison, Wisconsin, USA** | **23** | **1.86e-11** |
| **radical SAM** | **Acid Mine Drainage** | **Iron Mountain, California, USA** | **32** | **1.94e-11** |
| **radical SAM** | **Acid Mine Drainage** | **Iron Mountain, California, USA** | **32** | **1.97e-11** |
| **radical SAM** | **Acid Mine Drainage** | **Iron Mountain, California, USA** | **32** | **2.09e-11** |
| **radical SAM domain protein** | **Coastal** | **Galapagos Islands, Ecuador** | **26** | **2.11e-11** |
| **Radical SAM** | **Sewage Sludge** | **Madison, Wisconsin, USA** | **24** | **2.23e-11** |
| **radical SAM** | **Acid Mine Drainage** | **Iron Mountain, California, USA** | **26** | **2.40e-11** |
| Protein function | Metagenome | Location | % identity | e-value |
| **radical SAM** | **Hypersaline Lagoons** | **Galapagos Islands, Ecuador** | **24** | **2.91e-11** |
| **radical SAM** | **Acid Mine Drainage** | **Iron Mountain, California, USA** | **26** | **2.91e-11** |
| **radical SAM** | **Acid Mine Drainage** | **Iron Mountain, California, USA** | **26** | **3.01e-11** |
| **radical SAM** | **Acid Mine Drainage** | **Iron Mountain, California, USA** | **33** | **3.09e-11** |
| **radical SAM** | **Acid Mine Drainage** | **Iron Mountain, California, USA** | **26** | **3.35e-11** |
| **radical SAM** | **Acid Mine Drainage** | **Iron Mountain, California, USA** | **25** | **3.42e-11** |
| **radical SAM** | **Sewage Sludge** | **Brisbane, Queensland, Australia** | **23** | **3.72e-11** |
| **Radical SAM** | **Sewage Sludge** | **Madison, Wisconsin, USA** | **24** | **3.87e-11** |
| **radical SAM** | **Acid Mine Drainage** | **Iron Mountain, California, USA** | **27** | **3.91e-11** |
| **radical SAM** | **Acid Mine Drainage** | **Iron Mountain, California, USA** | **26** | **4.02e-11** |
| **radical SAM domain protein** | **Mangrove** | **Galapagos Islands, Ecuador** | **24** | **4.11e-11** |
| **radical SAM** | **Acid Mine Drainage** | **Iron Mountain, California, USA** | **27** | **4.23e-11** |
| **radical SAM** | **Acid Mine Drainage** | **Iron Mountain, California, USA** | **27** | **4.49e-11** |
| **radical SAM domain protein** | **Mangrove** | **Galapagos Islands, Ecuador** | **25** | **4.50e-11** |
| **radical SAM domain protein** | **Mangrove** | **Galapagos Islands, Ecuador** | **30** | **4.74e-11** |
| **radical SAM** | **Acid Mine Drainage** | **Iron Mountain, California, USA** | **27** | **5.08e-11** |
| **radical SAM** | **Acid Mine Drainage** | **Iron Mountain, California, USA** | **27** | **5.17e-11** |
| **radical SAM** | **Acid Mine Drainage** | **Iron Mountain, California, USA** | **27** | **6.32e-11** |
| **radical SAM domain protein** | **Surface soil** | **Minnesota, USA** | **30** | **6.37e-11** |
| **Radical SAM** | **Sewage Sludge** | **Madison, Wisconsin, USA** | **23** | **7.18e-11** |
| **radical SAM domain protein** | **Silicate Sediments** | **Mediterranean Sea, Italy** | **26** | **7.68e-11** |
| **radical SAM domain protein** | **Open Ocean** | **Sargasso Sea, Bermuda** | **29** | **9.15e-11** |
| **radical SAM** | **Sewage Sludge** | **Brisbane, Queensland, Australia** | **23** | **9.78e-11** |
| **radical SAM** | **Sewage Sludge** | **Brisbane, Queensland, Australia** | **22** | **1.20e-10** |
| **radical SAM domain protein** | **Silicate Sediments** | **Mediterranean Sea, Italy** | **31** | **1.24e-10** |
| **radical SAM** | **Hypersaline Lagoons** | **Galapagos Islands, Ecuador** | **29** | **1.24e-10** |
| **radical SAM domain protein** | **Open Ocean** | **Sargasso Sea, Bermuda** | **29** | **1.26e-10** |
| **radical SAM domain protein** | **Coastal** | **North American East Coast, USA** | **24** | **1.27e-10** |
| **radical SAM domain protein** | **Open Ocean** | **Sargasso Sea, Bermuda** | **29** | **1.34e-10** |
| **radical SAM domain protein** | **Silicate Sediments** | **Mediterranean Sea, Italy** | **28** | **1.34e-10** |
| **radical SAM** | **Acid Mine Drainage** | **Iron Mountain, California, USA** | **26** | **1.39e-10** |
| **radical SAM domain protein** | **Open Ocean** | **Sargasso Sea, Bermuda** | **29** | **1.41e-10** |
| **radical SAM domain protein** | **Open Ocean** | **Sargasso Sea, Bermuda** | **29** | **1.45e-10** |
| **radical SAM domain protein** | **Mangrove** | **Galapagos Islands, Ecuador** | **25** | **1.48e-10** |
| **radical SAM domain protein** | **Open Ocean** | **Sargasso Sea, Bermuda** | **26** | **1.53e-10** |
| **radical SAM** | **Acid Mine Drainage** | **Iron Mountain, California, USA** | **27** | **1.58e-10** |
| **radical SAM domain protein** | **Open Ocean** | **Sargasso Sea, Bermuda** | **31** | **1.60e-10** |
| **radical SAM** | **Hypersaline Lagoons** | **Galapagos Islands, Ecuador** | **25** | **1.71e-10** |
| **radical SAM domain protein** | **Open Ocean** | **Sargasso Sea, Bermuda** | **31** | **1.80e-10** |
| **radical SAM domain protein** | **Open Ocean** | **Sargasso Sea, Bermuda** | **31** | **1.88e-10** |
| **radical SAM domain protein** | **Open Ocean** | **Sargasso Sea, Bermuda** | **29** | **1.89e-10** |
| **radical SAM domain protein** | **Silicate Sediments** | **Mediterranean Sea, Italy** | **28** | **1.95e-10** |
| **radical SAM domain protein** | **Open Ocean** | **Sargasso Sea, Bermuda** | **30** | **1.97e-10** |
| **radical SAM domain protein** | **Open Ocean** | **Sargasso Sea, Bermuda** | **29** | **2.04e-10** |
| **radical SAM** | **Acid Mine Drainage** | **Iron Mountain, California, USA** | **24** | **2.54e-10** |
| **radical SAM domain protein** | **Open Ocean** | **Sargasso Sea, Bermuda** | **27** | **2.61e-10** |
| **radical SAM** | **Hypersaline Lagoons** | **Galapagos Islands, Ecuador** | **26** | **2.87e-10** |
| **radical SAM domain protein** | **Open Ocean** | **Sargasso Sea, Bermuda** | **25** | **2.96e-10** |
| **radical SAM domain protein** | **Open Ocean** | **Sargasso Sea, Bermuda** | **26** | **3.11e-10** |
| **radical SAM** | **Acid Mine Drainage** | **Iron Mountain, California, USA** | **26** | **3.35e-10** |
| **radical SAM** | **Acid Mine Drainage** | **Iron Mountain, California, USA** | **26** | **3.38e-10** |
| **radical SAM domain protein** | **Open Ocean** | **Sargasso Sea, Bermuda** | **27** | **3.41e-10** |
| **radical SAM domain protein** | **Open Ocean** | **Sargasso Sea, Bermuda** | **27** | **3.55e-10** |
| **radical SAM domain protein** | **Silicate Sediments** | **Mediterranean Sea, Italy** | **31** | **3.63e-10** |
| **radical SAM** | **Acid Mine Drainage** | **Iron Mountain, California, USA** | **26** | **3.70e-10** |
| **radical SAM domain protein** | **Open Ocean** | **Sargasso Sea, Bermuda** | **26** | **3.80e-10** |
| **radical SAM domain protein** | **Open Ocean** | **Sargasso Sea, Bermuda** | **26** | **3.83e-10** |
| **radical SAM** | **Sewage Sludge** | **Brisbane, Queensland, Australia** | **24** | **4.07e-10** |
| **radical SAM** | **Acid Mine Drainage** | **Iron Mountain, California, USA** | **26** | **4.13e-10** |
| **radical SAM domain protein** | **Open Ocean** | **Sargasso Sea, Bermuda** | **26** | **4.13e-10** |
| **radical SAM domain protein** | **Silicate Sediments** | **Mediterranean Sea, Italy** | **31** | **4.39e-10** |
| **radical SAM domain protein** | **Open Ocean** | **Sargasso Sea, Bermuda** | **26** | **4.49e-10** |
| **radical SAM domain protein** | **Silicate Sediments** | **Mediterranean Sea, Italy** | **31** | **4.54e-10** |
| **radical SAM domain protein** | **Open Ocean** | **Sargasso Sea, Bermuda** | **26** | **4.60e-10** |
| **radical SAM domain protein** | **Open Ocean** | **Sargasso Sea, Bermuda** | **26** | **4.64e-10** |
| **radical SAM domain protein** | **Open Ocean** | **Sargasso Sea, Bermuda** | **26** | **4.72e-10** |
| **radical SAM domain protein** | **Open Ocean** | **Sargasso Sea, Bermuda** | **26** | **5.09e-10** |
| **radical SAM domain protein** | **Open Ocean** | **Sargasso Sea, Bermuda** | **28** | **5.35e-10** |
| **radical SAM domain protein** | **Surface soil** | **Minnesota, USA** | **25** | **5.69e-10** |
| **radical SAM domain protein** | **Open Ocean** | **Sargasso Sea, Bermuda** | **26** | **6.87e-10** |
| **radical SAM** | **Acid Mine Drainage** | **Iron Mountain, California, USA** | **30** | **7.63e-10** |
| **radical SAM domain protein** | **Open Ocean** | **Sargasso Sea, Bermuda** | **27** | **7.91e-10** |
| **radical SAM domain protein** | **Open Ocean** | **Sargasso Sea, Bermuda** | **27** | **8.46e-10** |
| **radical SAM domain protein** | **Mangrove** | **Galapagos Islands, Ecuador** | **26** | **8.56e-10** |
| **radical SAM domain protein** | **Mangrove** | **Galapagos Islands, Ecuador** | **31** | **9.59e-10** |
| **radical SAM domain protein** | **Mangrove** | **Galapagos Islands, Ecuador** | **30** | **1.08e-9** |
| Protein function | Metagenome | Location | % identity | e-value |
| **radical SAM domain protein** | **Mangrove** | **Galapagos Islands, Ecuador** | **30** | **1.10e-9** |
| **radical SAM domain protein** | **Open Ocean** | **Sargasso Sea, Bermuda** | **30** | **1.15e-9** |
| **radical SAM domain protein** | **Open Ocean** | **Sargasso Sea, Bermuda** | **27** | **1.17e-9** |
| **Putative arylsulfatase regulatory protein** | **Harbor** | **Indian Ocean, Tanzania** | **28** | **1.20e-9** |
| **radical SAM domain protein** | **Open Ocean** | **Sargasso Sea, Bermuda** | **26** | **1.24e-9** |
| **Hypothetical protein** | **Coral Reef Atoll** | **Polynesia Archipelagos, French Polynesia** | **37** | **1.36e-9** |
| **radical SAM domain protein** | **Open Ocean** | **Sargasso Sea, Bermuda** | **26** | **1.49e-9** |
| **radical SAM domain protein** | **Open Ocean** | **Sargasso Sea, Bermuda** | **25** | **1.52e-9** |
| **radical SAM domain protein** | **Open Ocean** | **Sargasso Sea, Bermuda** | **25** | **1.56e-9** |
| **radical SAM domain protein** | **Open Ocean** | **Sargasso Sea, Bermuda** | **28** | **1.69e-9** |
| **radical SAM domain protein** | **Open Ocean** | **Sargasso Sea, Bermuda** | **26** | **1.73e-9** |
| **radical SAM domain protein** | **Open Ocean** | **Sargasso Sea, Bermuda** | **26** | **1.88e-9** |
| **radical SAM domain protein** | **Open Ocean** | **Sargasso Sea, Bermuda** | **26** | **2.01e-9** |
| **radical SAM domain protein** | **Open Ocean** | **Sargasso Sea, Bermuda** | **26** | **2.12e-9** |
| **radical SAM domain protein** | **Open Ocean** | **Sargasso Sea, Bermuda** | **26** | **2.19e-9** |
| **radical SAM domain protein** | **Surface soil** | **Minnesota, USA** | **33** | **2.26e-9** |
| **Fe-S oxidoreductases** | **Coastal** | **Galapagos Islands, Ecuador** | **36** | **7.62e-9** |
| **radical SAM** | **Hypersaline Lagoons** | **Galapagos Islands, Ecuador** | **30** | **7.98e-9** |
| **radical SAM** | **Acid Mine Drainage** | **Iron Mountain, California, USA** | **25** | **1.35e-8** |
| **radical SAM domain protein** | **Open Ocean** | **Sargasso Sea, Bermuda** | **26** | **1.58e-8** |
| **radical SAM domain protein** | **Open Ocean** | **Sargasso Sea, Bermuda** | **22** | **2.19e-8** |
| **radical SAM domain protein** | **Open Ocean** | **Sargasso Sea, Bermuda** | **22** | **2.21e-8** |
| **radical SAM** | **Hypersaline Lagoons** | **Galapagos Islands, Ecuador** | **29** | **2.36e-8** |
| **radical SAM domain protein** | **Open Ocean** | **Sargasso Sea, Bermuda** | **21** | **2.36e-8** |
| **radical SAM domain protein** | **Open Ocean** | **Sargasso Sea, Bermuda** | **21** | **2.42e-8** |
| **radical SAM domain protein** | **Open Ocean** | **Sargasso Sea, Bermuda** | **30** | **2.88e-8** |
| **radical SAM domain protein** | **Open Ocean** | **Sargasso Sea, Bermuda** | **30** | **3.47e-8** |
| **radical SAM domain protein** | **Open Ocean** | **Sargasso Sea, Bermuda** | **22** | **4.45e-8** |
| **molybdenum cofactor biosynthesis protein A** | **Open Ocean** | **Eastern Tropical Pacific, Panama** | **23** | **1.214** |
| **molybdenum cofactor biosynthesis protein A** | **Open Ocean** | **Eastern Tropical Pacific, Panama** | **23** | **1.838** |
| **radical SAM domain protein** | **Mangrove** | **Isabella Island, Galapagos Islands, Ecuador** | **31** | **3.047** |
| **radical SAM domain protein** | **Mangrove** | **Isabella Island, Galapagos Islands, Ecuador** | **23** | **3.704** |
| **Hypothetical** | **Hydrothermal vent** | **Eastern Pacific Ocean, International** | **39** | **5.99** |
| **radical SAM domain protein** | **Mangrove** | **Isabella Island, Galapagos Islands, Ecuador** | **23** | **7.934** |
